# A Prognostic Ferroptosis-Related lncRNAs Signature Associated With Immune Landscape and Radiotherapy Response in Glioma

**DOI:** 10.3389/fcell.2021.675555

**Published:** 2021-05-19

**Authors:** Jianglin Zheng, Zijie Zhou, Yue Qiu, Minjie Wang, Hao Yu, Zhipeng Wu, Xuan Wang, Xiaobing Jiang

**Affiliations:** ^1^Department of Neurosurgery, Union Hospital, Tongji Medical College, Huazhong University of Science and Technology, Wuhan, China; ^2^Department of Otolaryngology, Union Hospital, Tongji Medical College, Huazhong University of Science and Technology, Wuhan, China

**Keywords:** ferroptosis, long non-coding RNA, glioma, prognostic signature, immune landscape, chemoradiotherapy

## Abstract

Recent studies have demonstrated that long non-coding RNAs (lncRNAs) are implicated in the regulation of tumor cell ferroptosis. However, the prognostic value of ferroptosis-related lncRNAs has never been comprehensively explored in glioma. In this study, the transcriptomic data and clinical information of glioma patients were downloaded from TCGA, CGGA and Rembrandt databases. We identified 24 prognostic ferroptosis-related lncRNAs, 15 of which (SNAI3-AS1, GDNF-AS1, WDFY3-AS2, CPB2-AS1, WAC-AS1, SLC25A21-AS1, ARHGEF26-AS1, LINC00641, LINC00844, MIR155HG, MIR22HG, PVT1, SNHG18, PAXIP1-AS2, and SBF2-AS1) were used to construct a ferroptosis-related lncRNAs signature (FRLS) according to the least absolute shrinkage and selection operator (LASSO) regression. The validity of this FRLS was verified in training (TCGA) and validation (CGGA and Rembrandt) cohorts, respectively. The Kaplan-Meier curves revealed a significant distinction of overall survival (OS) between the high- and low-risk groups. The receiver operating characteristic (ROC) curves exhibited robust prognostic capacity of this FRLS. A nomogram with improved accuracy for predicting OS was established based on independent prognostic factors (FRLS, age, and WHO grade). Besides, patients in the high-risk group had higher immune, stroma, and ESTIMATE scores, lower tumor purity, higher infiltration of immunosuppressive cells, and higher expression of immune checkpoints. Patients in the low-risk group benefited significantly from radiotherapy, while no survival benefit of radiotherapy was observed for those in the high-risk group. In conclusion, we identified the prognostic ferroptosis-related lncRNAs in glioma, and constructed a prognostic signature which was associated with the immune landscape of glioma microenvironment and radiotherapy response.

## Introduction

Glioma is the most common primary malignancy in central nervous system, constituting approximately 80.1% of all primary malignant brain tumors (Ostrom et al., 2020). The survival rate of glioma patients is known to decrease with increased World Health Organization (WHO) grade (Louis et al., 2016). Glioblastoma (GBM; WHO grade IV), the most malignant type of glioma, has a dismal prognosis with a median overall survival (OS) of approximately 8 months and a 5-year survival rate of 7.2%, regardless of the treatment received (Ostrom et al., 2020). It has become a disturbing issue for neurosurgeons and oncologists that the prognosis of most patients with glioma has not improved significantly using existing therapeutic options (Ding et al., 2017). A major clinical challenge is the high heterogeneity of glioma, which leads to the inconsistencies in therapeutic response and prognosis (van den Bent et al., 2009). Hence, identifying novel biomarkers for the prediction of therapeutic response and prognosis is of great clinical significance for glioma patients.

Ferroptosis, a novel type of programmed cell death (PCD), differs from apoptosis and autophagy in its unique mechanism that iron-dependent accumulation of reactive oxygen species (ROS) and irresistible lipid peroxidation result in the cell death (Dixon et al., 2012; Cao and Dixon, 2016). Recently, increasing evidences have verified that ferroptosis correlates with progression and therapeutic response of glioma (Lang et al., 2019; Tang R. et al., 2020; Xu Y. et al., 2020). The dysregulation of ferroptosis-related genes (for example, GPX4, SLC7A11, and ACSL4) was found capable of protecting glioma cells from ferroptosis (Wang et al., 2018; Cheng et al., 2020; Zhang et al., 2020). Erastin, a ferroptosis inducer, has been reported to have temozolomide (TMZ)-sensitizing effect on glioma cells (Chen et al., 2015). Therefore, we hypothesized that ferroptosis-related biomarkers hold immense potential toward the prediction of therapeutic response and prognosis in glioma patients.

The long non-coding RNAs (lncRNAs), defined as a subclass of non-coding RNAs with a length of >200 nucleotides (Alexander et al., 2010), have been shown to involve in a broad array of tumor biological behavior (Fatica and Bozzoni, 2014). Recent studies indicated that dysregulation of specific lncRNAs was inextricably linked with the ferroptosis process of malignant tumors (Yan et al., 2015; Wu et al., 2020). It was reported that upregulation of lncRNA LINC00336 could inhibit ferroptosis in lung cancer (Wang M. et al., 2019). Another study revealed that upregulation of lncRNA LINC00618 promote vincristine-induced ferroptosis in human leukemia (Wang et al., 2020). However, the role of lncRNAs in ferroptosis process of glioma remains obscure. The value of ferroptosis-related lncRNAs as prognostic biomarkers for glioma patients has never been systematically evaluated.

Here, owe to the great advances of genome sequencing technology and bioinformatics, we systematically evaluated the identified ferroptosis-related lncRNAs in glioma by integrating The Cancer Genome Atlas (TCGA), Chinese Glioma Genome Atlas (CGGA), and Rembrandt databases. A prognostic ferroptosis-related lncRNAs signature (FRLS) based on 15 ferroptosis-related lncRNAs was constructed for glioma patients and its correlations with immune landscape and the efficacy of chemoradiotherapy were also investigated. We aimed to provide a new strategy for the prediction of prognosis and treatment efficacy in glioma patients.

## Materials and Methods

### Patient Data Collection

The RNA-seq transcriptome data and clinical information of glioma patients were extracted from TCGA^1^, CGGA^2^, and Rembrandt^3^ databases. Patients with missing survival data or OS <30 days, or without definitive histopathological diagnosis were excluded. Eventually, a total of 1,904 glioma patients were included in the subsequent analyses. The TCGA dataset (*n* = 611) served as a training cohort. The CGGA dataset (*n* = 966) and the Rembrandt dataset (*n* = 327) were used as validation cohorts. The RNA-seq transcriptome data of TCGA and CGGA datasets were downloaded in the format of fragments per kilobase of exon model per million mapped reads (FPKM) normalized, whereas that of Rembrandt dataset was normalized microarray format. The clinicopathological characteristics of all included patients were summarized in Table 1.

**TABLE 1:** Characteristics of glioma patients in training and validation cohorts.

Characteristics		Training cohort	Validation cohorts	
		
		TCGA (*n* = 611)	CGGA (*n* = 966)	Rembrandt (*n* = 327)
Age (years)	<= 50	349	698	127
	>50	262	268	157
	NA	0	0	43
Gender	Female	255	399	89
	Male	356	567	147
	NA	0	0	91
2016 WHO Classification	A, IDH-mutant	39	110	NA
	A, IDH-wild type	15	43	NA
	AA, IDH-mutant	78	121	NA
	AA, IDH-wild type	33	82	NA
	O, IDH-mutant and 1p/19q-codel	29	84	NA
	AO, IDH-mutant and 1p/19q-codel	22	67	NA
	GBM, IDH-mutant	69	84	NA
	GBM, IDH-wild type	88	280	NA
	NOS	238	95	NA
Grade	II	215	270	69
	III	236	322	71
	IV	160	374	187
IDH status	Mutant	370	499	NA
	Wild type	234	418	NA
	NA	7	49	NA
1p19q codeletion	Codel	146	199	NA
	Non-codel	459	696	NA
	NA	6	71	NA
MGMT promoter status	Methylated	425	454	NA
	Unmethylated	154	359	NA
	NA	32	153	NA
TMZ chemotherapy	Yes	436	668	NA
	No	94	265	NA
	NA	82	33	NA
Radiotherapy	Yes	119	740	NA
	No	94	192	NA
	NA	398	34	NA

*A, astrocytoma; AA, anaplastic astrocytoma; O, oligodendroglioma; AO, anaplastic oligodendroglioma; GBM, glioblastoma; NOS, not otherwise specified.*

### Identification of Prognostic Ferroptosis-Related lncRNAs

A total of 60 ferroptosis-related genes were collected according to the published studies (Stockwell et al., 2017; Hassannia et al., 2019; Liang et al., 2020; Liu H.J. et al., 2020). Based on the lncRNA annotation file of Genome Reference Consortium Human Build 38 (GRCh38) downloaded from the GENCODE website^4^, we extracted the expression data of 14,142 lncRNAs in the TCGA dataset and 1,005 lncRNAs in the CGGA dataset. Pearson correlation analysis between the ferroptosis-related genes and lncRNAs was first implemented to identify the ferroptosis-related lncRNAs (| R| > 0.5 and *p* < 0.001) in the TCGA and CGGA cohorts, respectively. Univariate Cox regression analysis was subsequently performed for prognostic identification (*p* < 0.05). The prognostic ferroptosis-related lncRNAs shared by two cohorts were considered as eligible.

### Construction and Validation of the Prognostic FRLS

The prognostic ferroptosis-related lncRNAs were incorporated into the LASSO regression, which was performed within the TCGA cohort by using the R package “glmnet” (Friedman et al., 2010). The prognostic FRLS was consequently constructed by selecting the optimal penalty parameter λ correlated with the minimum 10-fold cross-validation. The calculation formula of risk score is shown below:

Risk score=$\sum_{i=1}^{n}Coef_{i}^{*}x_{i}$

where *x*_*i*_ d *Coef*_*i*_ present the expression level of each selected lncRNA and corresponding coefficient, respectively. The median risk score was used as the cut-off value for the high/low-risk grouping of patients. The Kaplan-Meier curve with log-rank test was generated by using the R package “survminer” for the comparison of OS between the high- and low-risk groups. The ROC curve analysis was utilized to evaluate the prediction accuracy of FRLS via the R package “timeROC.” All the validations were performed simultaneously in the training and validation cohorts.

### Establishment and Evaluation of a Nomogram

By employing the R package “rms,” “regplot,” and “Hmisc,” a nomogram was established based on the independent prognostic factors in the TCGA cohort, which were determined through univariate and multivariate Cox regression analyses. The availability of this nomogram was evaluated by the C-index (Harrell et al., 1996) and calibration curve. The ROC analysis was also performed to assess the accuracy of the nomogram for OS prediction.

### Functional Enrichment Analysis

Based on the expression levels of 60 ferroptosis-related genes, PCA was performed using the R package “scatterplot3d” to explore the potential differences in ferroptosis sensitivity between the high- and low-risk groups. The DEGs between the high- and low-risk groups were identified (| log2FC| > 2 and adjusted *p* < 0.05), and functionally annotated by the Gene Ontology (GO) and the Kyoto Encyclopedia of genes and Genomes (KEGG) pathway analyses via the R package “clusterProfiler,” “org.Hs.eg.db,” and “enrichplot.”

### Evaluation of the Immune Landscape

The immune scores and stromal scores of glioma patients were calculated using the ESTIMATE algorithm via the R package “estimate” (Yoshihara et al., 2013). The abundance of 22 immune cells was calculated through CIBERSORT algorithm with 1,000 permutations (Newman et al., 2015). Patients with CIBERSORT *p* ≥ 0.05 were excluded from the subsequent analysis. We evaluated the differences between the high- and low-risk groups in the abundance of 22 immune cells and the expression levels of immune-related molecules. Besides, we applied Pearson correlation analysis to calculate the correlation between risk scores and the expression levels of immune cell markers, which were well elucidated by previous studies (Danaher et al., 2017; Tan et al., 2020).

### Tissue Samples and Quantitative Real-Time Polymerase Chain Reaction (qRT-PCR)

All tissue samples were collected from the Neurosurgery Department of Wuhan Union Hospital, which was approved by the Medical Ethics Committee of the hospital. We acquired the informed consent from each involved patient. A total of 10 glioma tissue samples (4 WHO grade II, 2 WHO grade III, and 4 GBM) were obtained from glioma patients who underwent tumor resection between October 2020 and February 2021. Six non-tumor brain tissues were obtained from patients with brain tissue resection due to craniocerebral injury from May 2020 to February 2021. The locations of tissues were summarized in Supplementary Table 1. Fresh tumor and non-tumor tissues were snap frozen in liquid nitrogen. Total RNA was extracted from tissues using RNAiso Plus (Takara 9109). Referring to the manufacturer instruction, cDNA was synthesized by reverse transcription using reverse transcription kit (Takara RR036A). The qRT-PCR analysis was further performed on the LightCycler 480 Real-Time PCR system using TB Green Premix Ex Taq^TM^ II (Takara RR820A). All expression data was normalized to GAPDH as an internal control using the 2^–ΔΔ*Ct*^ method. All primers used were chemically synthesized by Sangon Biotech (Sangon Biotech, Shanghai, China). The primer sequences were listed in Supplementary Table 2.

### Statistical Analysis

The preprocessing of RNA-seq transcriptome data was performed using PERL programming language (version 5.32.0). The R software (version 4.0.2) were applied for all statistical analyses and graph visualization. The Chi-square test was executed for the comparison of categorical variables between the high- and low-risk groups. The Student’s *t*-test or one-way ANOVA test was utilized to compare the continuous variables with normal distribution (including risk score, immune score, stromal score, ESTIMATE score and tumor purity) between two groups or more than two groups. The Wilcox test was performed to determine the differences between the high- and low-risk groups in the abundance of 22 immune cells, the expression level of immune checkpoints and ferroptosis-related lncRNAs. The non-parametric test was used to compare the expression level of selected ferroptosis-related lncRNAs between glioma tissues and non-tumor brain tissues. Two-tailed *p* < 0.05 was considered statistically significant.

## Results

### Identification of Prognostic Ferroptosis-Related lncRNAs in Glioma Patients

**FIGURE 1:**
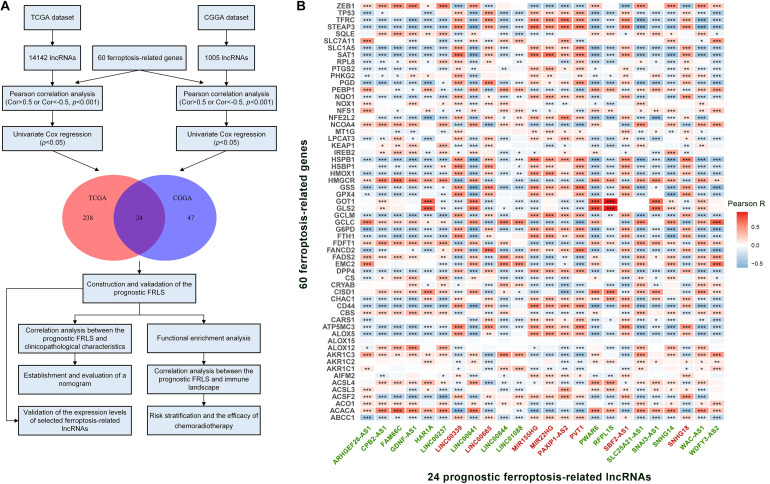
**(A)** The flow diagram of the research process. **(B)** The correlation between 60 ferroptosis-related genes and 24 prognostic ferroptosis-related lncRNAs in the TCGA cohort. The ferroptosis-related lncRNAs with green font are protective factors for survival and the ferroptosis-related lncRNAs with red font are risk factors for survival. *p < 0.05, **p < 0.01, and ***p < 0.001.

A complete flow diagram of the research process was illustrated in Figure 1A. After performing the match between the ENSEMBL ID and the lncRNA annotation file, we obtained 14,142 and 1,005 lncRNAs in TCGA and CGGA datasets, respectively. Besides, 60 ferroptosis-related genes were sorted out according to the published literature. A ferroptosis-related lncRNA would be identified if it was significantly correlated with one or more ferroptosis-related genes (| R| > 0.5 and *p* < 0.001). We obtained 427 and 288 ferroptosis-related lncRNAs in TCGA and CGGA datasets, respectively. Combined with Univariate Cox regression analyses, we screened out 24 prognostic ferroptosis-related lncRNAs shared by two datasets. The co-expression relationship between the 24 lncRNAs and 60 ferroptosis-related genes was shown in Figure 1B. Among these 24 lncRNAs, 16 were protective factors and 8 were risky factors for prognosis.

### Construction and Validation of the Prognostic FRLS

**FIGURE 2:**
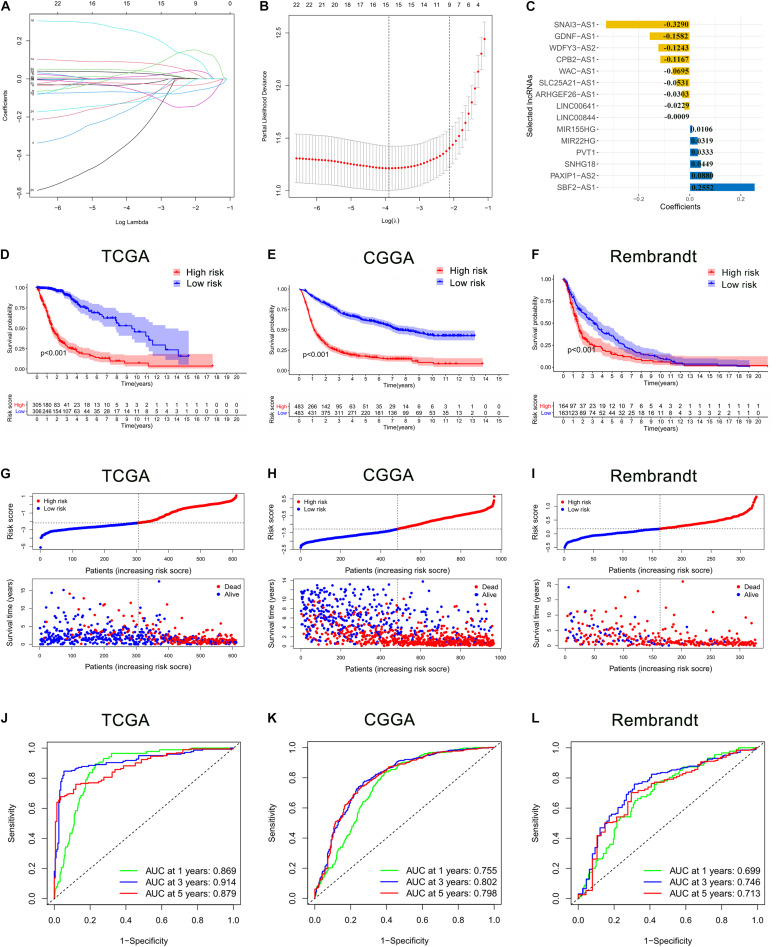
Construction and validation of the prognostic ferroptosis-related lncRNAs signature (FRLS). **(A,B)** The least absolute shrinkage and selection operator (LASSO) regression was performed with the minimum criteria. **(C)** LASSO coefficients of 15 selected ferroptosis-related lncRNAs. **(D–F)** The Kaplan-Meier curves for survival in the TCGA, CGGA and Rembrandt cohorts. **(G–I)** The distribution plots of the risk score and survival status in the TCGA, CGGA and Rembrandt cohorts. **(J–L)** The receiver operating characteristic (ROC) curve analyses of the prognostic FRLS in predicting 1-, 3-, and 5-year overall survival (OS) in the TCGA, CGGA, and Rembrandt cohorts.

These 24 ferroptosis-related lncRNAs were incorporated into the least absolute shrinkage and selection operator (LASSO) regression in the TCGA cohort. As a result, 15 ferroptosis-related lncRNAs stood out for the construction of the prognostic FRLS, including SNAI3-AS1, GDNF-AS1, WDFY3-AS2, CPB2-AS1, WAC-AS1, SLC25A21-AS1, ARHGEF26-AS1, LINC00641, LINC00844, MIR155HG, MIR22HG, PVT1, SNHG18, PAXIP1-AS2, and SBF2-AS1 (Figures 2A–C). The survival analyses of these 15 ferroptosis-related lncRNAs were illustrated in Supplementary Figure 1. Then, the risk score for each glioma patient was calculated by summing the product of the expression level of each selected ferroptosis-related lncRNA and corresponding coefficient. Then, glioma patients were stratified into the high- and low-risk groups using the median risk score as the cut-off value. In the TCGA cohort, the Kaplan-Meier curve suggested that the OS of patients in the low-risk group was significantly longer than that of patients in the high-risk group (*p* < 0.001; Figure 2D). The distribution plot of the risk score and survival status showed that the higher the risk score, the more deaths of glioma patients (Figure 2G). A satisfactory prediction performance of FRLS was confirmed by the area under the receiver operating characteristic (ROC) curve (AUC) for 1-, 3,- and 5-year OS (AUC = 0.869, 0.914, and 0.879, respectively; Figure 2J).

To determine whether the prognostic significance of FRLS remained in other populations, the same analyses were performed in the CGGA cohort and the Rembrandt cohort. In accord with the findings in the TCGA cohort, patients in the low-risk group had better survival outcomes than patients in the high-risk group (Figures 2E,F,H,I). The AUCs for 1-, 3-, and 5-year OS in the CGGA cohort were 0.755, 0.802, and 0.798, respectively (Figure 2K), and in the Rembrandt cohort were 0.699, 0.746, and 0.713, respectively (Figure 2L). All results agreed that the prognostic FRLS could accurately and stably predict the survival outcome of glioma patients.

### Correlation Analysis Between the Prognostic FRLS and Clinicopathological Characteristics

**FIGURE 3:**
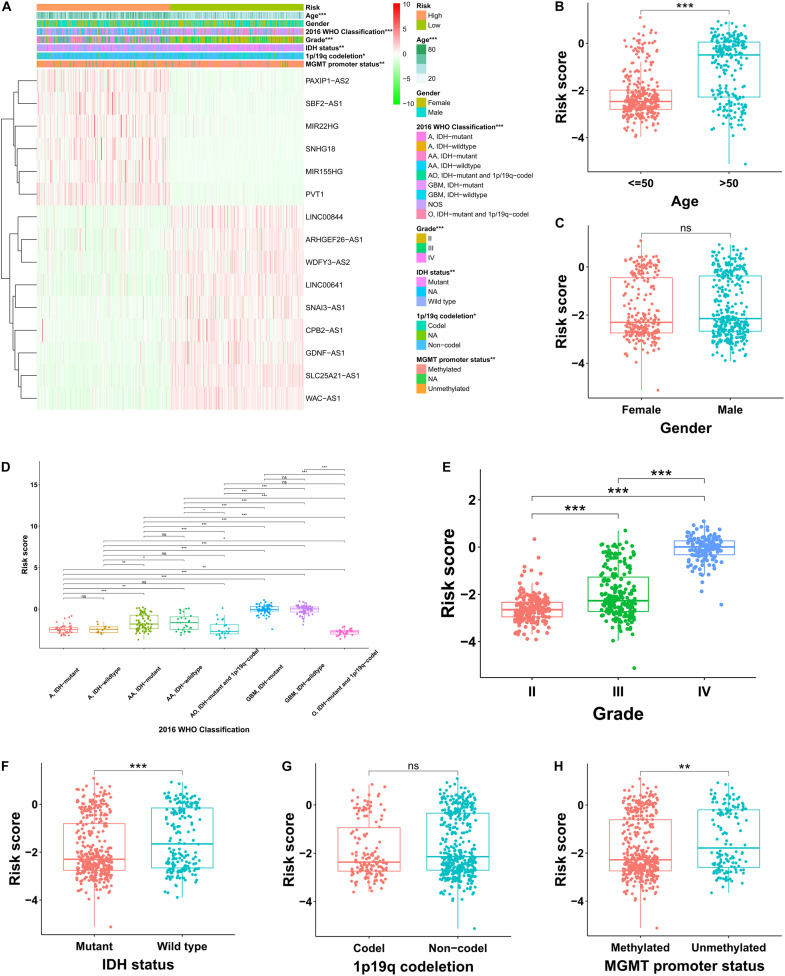
Correlation analysis between the prognostic FRLS and clinicopathological characteristics in the TCGA cohort. **(A)** A heatmap showing the distribution of clinicopathological characteristics and expression levels of 15 selected ferroptosis-related lncRNAs in the high- and low-risk groups. **(B–H)** Different levels of risk scores in glioma patients stratified by age, gender, 2016 WHO classification, grade, IDH status, 1p19q codeletion and MGMT promoter status. A, astrocytoma; AA, anaplastic astrocytoma; O, oligodendroglioma; AO, anaplastic oligodendroglioma; GBM, glioblastoma; NOS, not otherwise specified. *p < 0.05, **p < 0.01, ***p < 0.001, and ^*ns*^ No significance.

In the TCGA cohort, the risky lncRNAs involved in the construction of FRLS were up-regulated in the high-risk group and the protective lncRNAs were up-regulated in the low-risk group (Figure 3A). As the WHO grade increased, we observed significant increase in the expression levels of risky lncRNAs, whereas decrease in the expression levels of protective lncRNAs (Supplementary Figures 2A,B). Moreover, significant differences were observed between the two risk subgroups with respect to age, 2016 WHO classification, grade, IDH status, 1p19q codeletion and MGMT promoter status (Supplementary Tables 3–5). We also compared the levels of risk score between patients stratified by various clinicopathological characteristics. In the TCGA cohort, glioma patients with the clinicopathological characteristics of age >50 years, more malignant type of 2016 WHO classification, higher grade, IDH wild type, and MGMT promoter unmethylated showed significantly higher levels of risk score, while no risk score differences were observed between patients satisfied by gender and 1p19q codeletion (Figures 3B–H). Interestingly, the risk score of glioma patients in the CGGA cohort was elevated not only in the age >50 years, more malignant type of 2016 WHO classification, higher grade, IDH wild type, and MGMT promoter unmethylated subgroups, but also in the male and 1p19q non-codel patients (Supplementary Figures 3A–G). In the Rembrandt cohort, the clinicopathological characteristics of age >50 years and higher grade were associated with higher levels of risk score, whereas no significantly association between risk score and gender was recognized (Supplementary Figures 3H–J). To determine whether the clinicopathological characteristics would weaken the prediction accuracy of the prognostic FRLS, we performed subgroup survival analyses in three cohorts. The results showed that patients with high-risk score had worse survival outcomes than those with low-risk score in all subgroups, except for the grade IV subgroup in the TCGA cohort (Supplementary Figures 4A–C).

### Establishment and Evaluation of a Nomogram Based on Independent Prognostic Factors for OS

**FIGURE 4:**
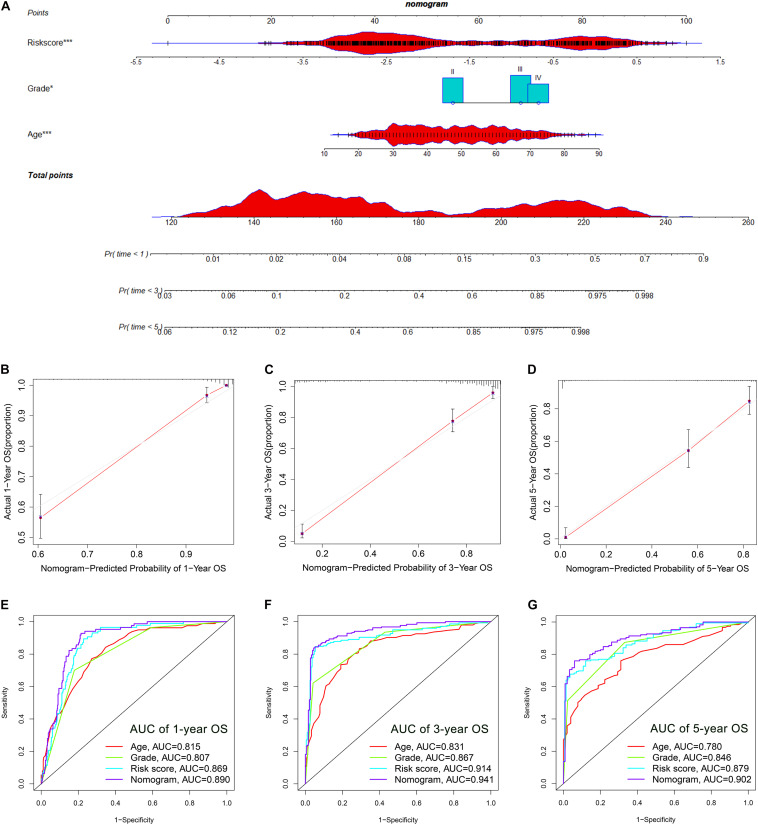
Establishment and evaluation of a nomogram in the TCGA cohort. **(A)** Nomogram based on FRLS, age and WHO grade. **(B–D)** Calibration curves showing the concordance between predicted and observed 1-, 3-, and 5-year overall survival (OS). **(E–G)** The receiver operating characteristic (ROC) curve analyses of the nomogram in predicting 1-, 3-, and 5-year OS. *p < 0.05, and ***p < 0.001.

The OS-related factors identified by univariate Cox regression analyses were subsequently analyzed using multivariate Cox regression. In the TCGA, CGGA, and Rembrandt cohorts, the FLRS-based risk score was always an independent prognostic factor after adjusted for other clinicopathological characteristics (Table 2 and Supplementary Table 6). Subsequently, we established a nomogram using these independent prognostic factors (Age, Grade, and Risk score) in the TCGA cohort (Figure 4A). The internal evaluation was initially performed. The concordance index (C-index) was 0.853 and the calibration plots demonstrated an excellent match between the actual and nomogram-predicted probability of 1-, 3-, and 5-year OS (Figures 4B–D). This nomogram exhibited the highest accuracy in predicting 1-, 3-, and 5-year OS (AUC = 0.890, 0.941, and 0.902, respectively) in comparison to other independent prognostic factors (Figures 4E–G). In the same way, the external evaluation of this nomogram was conducted in the CGGA cohort. The C-index was 0.841 and the calibration plots illustrated a satisfactory match between the actual and nomogram-predicted probability of 1-, 3-, and 5-year OS (Supplementary Figures 5A–C). Additionally, the accuracy of this nomogram in predicting 1-, 3-, and 5-year OS remained highest (AUC = 0.771, 0.833, and 0.828, respectively, Supplementary Figures 5D–F). It was of great significance to clinical practice that the nomogram had the potential to act as a quantitative instrument to predict the survival outcomes for glioma patients.

**TABLE 2:** Univariate and multivariate Cox analyses in the TCGA, CGGA and Rembrant cohorts.

Variables	Univariate analysis		Multivariate analysis	
	HR (95% CI)	*p*-value	HR (95% CI)	*p*-value
***TCGA***				
Age (Continuous)	1.066 (1.056–1.077)	<0.001	1.030 (1.018–1.042)	<0.001
Gender (Male vs. Female)	1.269 (0.976–1.651)	0.076	−	−
Grade (Continuous: IV, III, and II)	4.870 (3.957–5.994)	<0.001	1.429 (1.029–1.985)	0.033
IDH (Mutant vs. wild type)	0.661 (0.511–0.854)	0.002	0.718 (0.497–1.039)	0.079
1p/19q (Codel vs. Non-codel)	0.856 (0.629–1.166)	0.324	−	−
MGMT (Methylated vs. Unmethylated)	0.657 (0.497–0.869)	0.003	1.257 (0.846–1.868)	0.258
Risk score (Continuous)	2.993 (2.639–3.395)	<0.001	2.028 (1.633–2.519)	<0.001
***CGGA***				
Age (Continuous)	1.024 (1.016–1.032)	< 0.001	1.007 (1.000–1.014)	0.054
Gender (Male vs. Female)	1.072 (0.894–1.286)	0.453	−	−
Grade (Continuous: IV, III, and II)	2.712 (2.380–3.091)	<0.001	1.955 (1.680–2.275)	<0.001
IDH (Mutant vs. wild type)	0.340 (0.282–0.409)	<0.001	0.736 (0.550–0.983)	0.038
1p/19q (Codel vs. Non-codel)	0.237 (0.176–0.319)	<0.001	0.426 (0.308–0.591)	<0.001
MGMT (Methylated vs. Unmethylated)	0.807 (0.674–0.965)	0.019	0.912 (0.758–1.098)	0.329
Risk score (Continuous)	3.083 (2.678–3.550)	<0.001	1.926 (1.526–2.432)	<0.001
***Rembrandt***				
Gender (Male vs. Female)	1.032 (0.767–1.390)	0.834	−	−
Grade (Continuous: IV, III, and II)	1.699 (1.432–2.016)	<0.001	1.540 (1.290–1.839)	<0.001
Risk score (Continuous)	3.280 (2.261–4.758)	<0.001	2.435 (1.654–3.585)	<0.001

### Functional Enrichment Analyses

**FIGURE 5:**
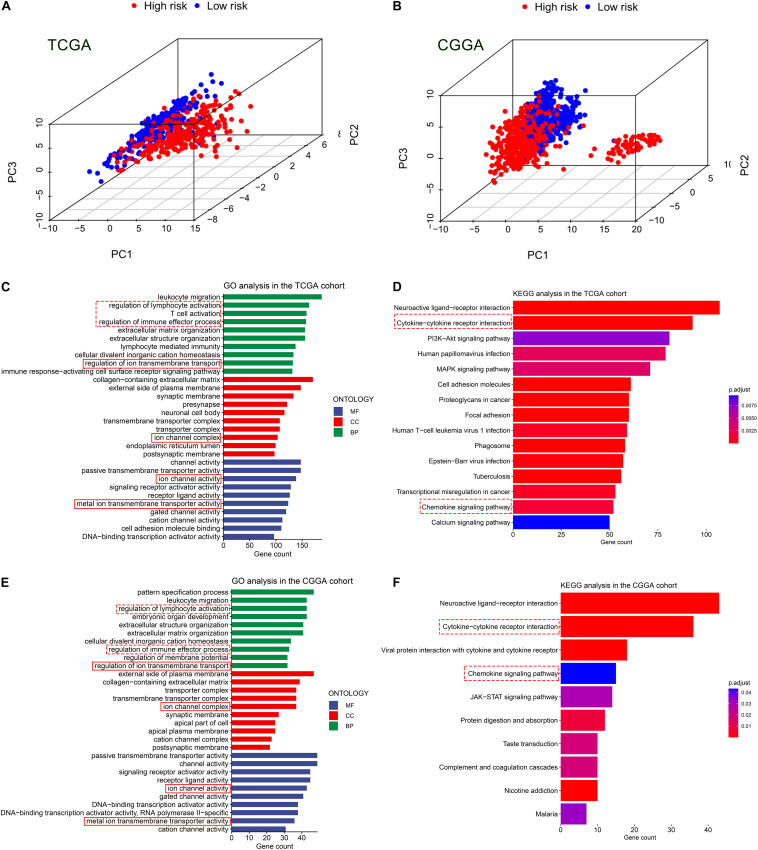
Principal component analyses (PCA) and representative results of functional enrichment analyses in the TCGA and CGGA cohorts. **(A,B)** PCA showing the distribution differences between the high- and low-risk groups. **(C,D)** Go analysis and KEGG analysis in the TCGA cohort. **(E,F)** Go analysis and KEGG analysis in the CGGA cohort. The dashed line boxes highlighted the immune-related biological processes or pathways. The solid line boxes highlighted the iron transport-related functions.

Based on the expression value of 60 ferroptosis-related genes, we first ran Principal Component Analyses (PCA) in the TCGA and CGGA cohorts. The prominent and stable distribution differences were observed between the high- and low-risk subgroups in both cohorts (Figures 5A,B). These may reflect, at least in part, the differences in the ferroptosis sensitivity between the two risk subgroups. We further performed functional enrichment analyses to characterize the biological functions of differentially expressed genes (DEGs) between the two risk subgroups. A total of 5,932 and 1,140 DEGs were identified in the TCGA cohort and CGGA cohort, respectively. Expectedly, the GO analyses in both cohorts revealed significant enrichment of iron transport-related functions, including regulation of ion transmembrane transport, ion channel complex, ion channel activity, and metal ion transmembrane transporter activity (Figures 5C,E). Interestingly, the DEGs were also enriched in several immune-related biological processes, for instances, regulation of lymphocyte activation, T cell activation and regulation of immune effector process (Figures 5C,E). Then, the KEGG pathway analyses similarly exhibited the significant enrichment of immune-related pathways in both cohorts, including cytokine-cytokine receptor interaction and chemokine signaling pathway (Figures 5D,F).

### Correlation of the Prognostic FRLS With the Immune Landscape of Glioma Microenvironment

**FIGURE 6:**
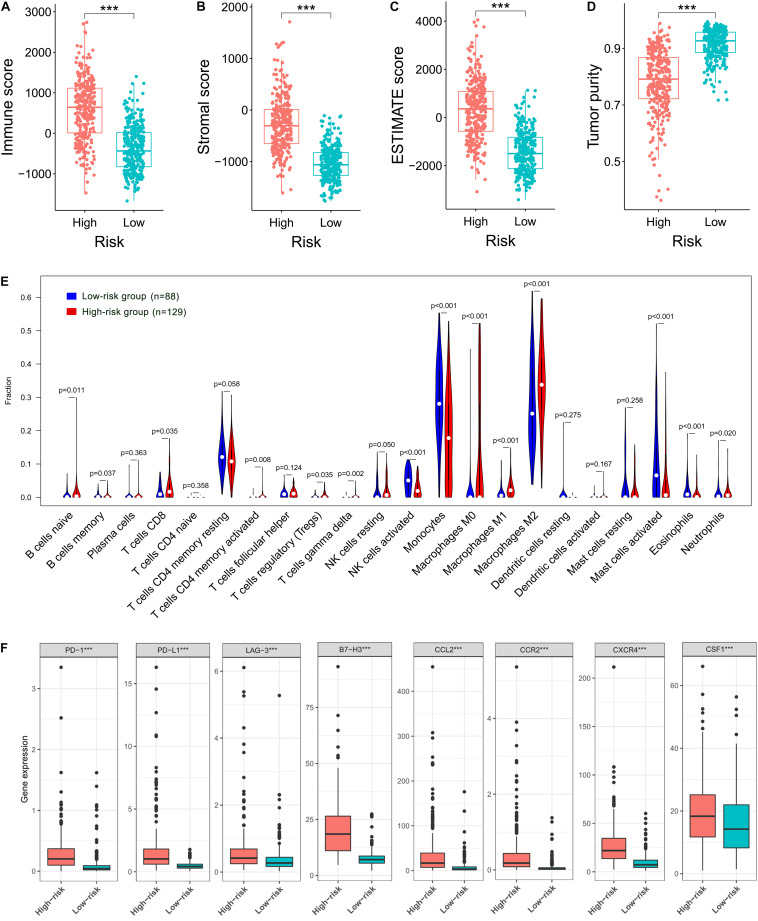
Correlation of the prognostic FRLS with the immune landscape of glioma microenvironment in the TCGA cohort. **(A–D)** Comparison of immune scores, stromal scores, ESTIMATE scores and tumor purity between the high- and low-risk groups. **(E)** The abundance of 22 immune cells in the high- and low-risk groups. A total of 394 patients with CIBERSORT *p* ≥ 0.05 were excluded. **(F)** The expression levels of immune checkpoints and macrophage associated molecules in the high- and low-risk groups. ***p < 0.001.

Given the results that the DEGs were enriched in the immune-related functions, we further investigated the correlation of the prognostic FRLS with the immune landscape of glioma microenvironment. In the TCGA cohort, the high-risk group showed significantly higher immune, stroma and ESTIMATE scores and lower tumor purity than the low-risk group (Figures 6A–D). Moreover, different extent of immune cell infiltrations was observed in the high-risk group with lower abundance of activated NK cells, monocytes, activated mast cells, and eosinophils, but higher abundance of naive B cells, memory B cells, CD8 + T cells, CD4 + memory activated T cells, regulatory T cells, gamma delta T cells, M0-type macrophages, M1-type macrophages, M2-type macrophages, and neutrophils (Figure 6E). Correlation analyses of the risk score with immune cell markers provided a more reliable confirmation of the differences between the two risk subgroups in the abundance of several immune cells (Supplementary Figures 6A–H). In addition, the immune checkpoints (PD-1, PD-L1, LAG-3, and B7-H3) and macrophage associated molecules (CCL2, CCR2, CXCR4, and CSF1) were up-regulated in the high-risk group (Figure 6F). These differential analyses between the two risk subgroups were carried out similarly in the CGGA cohort, and largely consistent findings were observed (Supplementary Figures 7A–F).

### Risk Stratification and the Efficacy of Chemoradiotherapy

**FIGURE 7:**
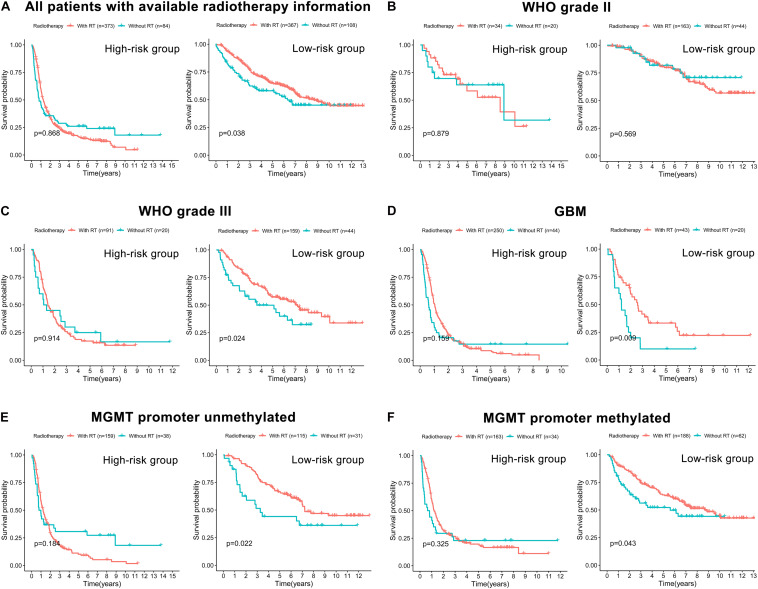
The correlation between FRLS-based risk stratification and the efficacy of radiotherapy in the CGGA cohort. **(A)** The Kaplan-Meier curves for patients with or without radiotherapy in the high- and low-risk groups. **(B–F)** The Kaplan-Meier curves for WHO grade II **(B)**, WHO grade III **(C)**, GBM **(D)**, MGMT promoter unmethylated **(E),** and MGMT promoter methylated **(F)** patients with or without radiotherapy in the high- and low-risk groups.

The correlation between the prognostic FRLS and the efficacy of chemoradiotherapy was investigated. In both CGGA and TCGA cohorts, the FRLS-based risk stratification was not correlated with the efficacy of TMZ treatment (Supplementary Figures 8, 9). Conversely, the survival benefit of radiotherapy was significant for patients in the low-risk group, while no significant survival benefit of radiotherapy was observed for those in the high-risk group (Figure 7A and Supplementary Figure 10A). Further analyses were performed in the subgroups stratified by two features, WHO grades and MGMT promoter status, which have reference significance for the choice of clinical treatment options. In the CGGA cohort, there were no significant survival differences between WHO grade II patients with or without radiotherapy in both high- and low-risk groups (Figure 7B). For the subgroups of WHO grade III, GBM, MGMT promoter unmethylated and MGMT promoter methylated, the survival benefit of radiotherapy remained significant in the low-risk group, and no survival benefit of radiotherapy was observed for those in the high-risk group (Figures 7C–F). In the TCGA cohort, similar results were noted in the subgroups of WHO grade III and MGMT promoter methylated, but not in the subgroups of WHO grade II and MGMT promoter unmethylated (Supplementary Figures 10B–E). The subgroup analysis was not conducted in GBM patients due to the limitation of available data. Accordingly, patients in the low-risk group were more likely to obtain a survival benefit from radiotherapy than those in the high-risk group.

### Validation of the Expression Levels of Selected Ferroptosis-Related lncRNAs

**FIGURE 8:**
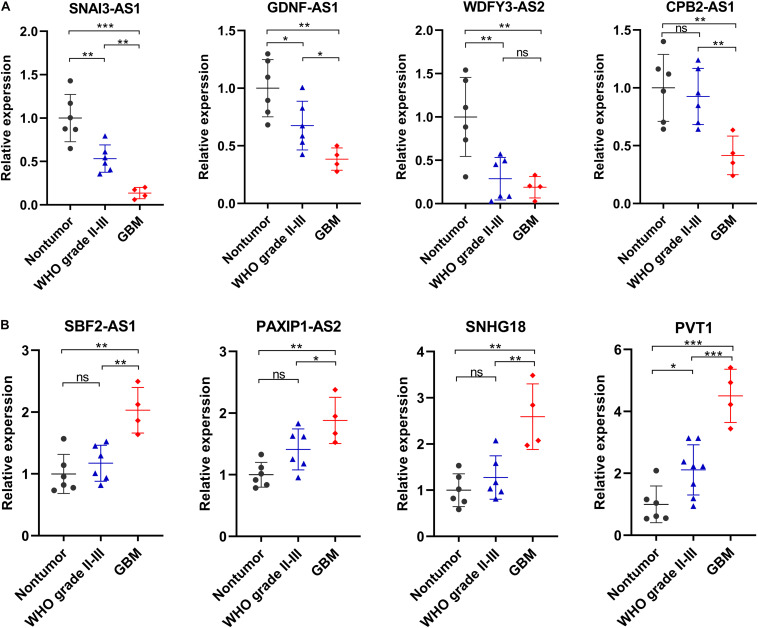
Validation of the expression levels of selected ferroptosis-related lncRNAs in in 6 non-tumor brain tissues and 10 glioma tissues. **(A)** Expression analysis of 4 protective lncRNAs (SNAI3-AS1, GDNF-AS1, WDFY3-AS2, and CPB2-AS1). **(B)** Expression analysis of 4 risky lncRNAs (SBF2-AS1, PAXIP1-AS2, SNHG18, and PVT1). *p < 0.05, **p < 0.01, ***p < 0.001, and ^*ns*^ No significance.

We selected eight ferroptosis-related lncRNAs for validation, whose LASSO coefficients ranked top four among protective lncRNAs and risky lncRNAs, respectively. By using RT-qPCR assay, we detected their expression levels in 6 non-tumor brain tissues and 10 glioma tissues (4 WHO grade II, 2 WHO grade III and 4 GBM). Compared with non-tumor brain tissues, the expression levels of SNAI3-AS1, GDNF-AS1, WDFY3-AS2, and CPB2-AS1 showed an overall downward trend (Figure 8A), and the expression level of SBF2-AS1, PAXIP1-AS2, SNHG18, and PVT1 showed an overall upward trend in glioma tissues (Figure 8B). Except for WDFY3-AS2, the difference in expression levels of these lncRNAs was also significant between WHO grade II-III and GBM tissues.

## Discussion

Induction of PCD was considered as the most promising antitumor mechanism. Ferroptosis, a non-apoptotic form of PCD, has recently emerged as a topic of intensive research in the field of tumorigenesis and therapies. It has been reported that ferroptosis-related biomarkers are robust predictors of prognosis and antitumor efficacy for cancers (Shi et al., 2019; Liang et al., 2020; Tang B. et al., 2020). Nevertheless, previous studies only focused on the ferroptosis-related genes encoding proteins involving in the regulation of ferroptosis. Considering the increasing evidences that lncRNAs play a key role in ferroptosis process through epigenetic regulation, we believe it is necessary to comprehensively evaluate the prognostic significance of ferroptosis-related lncRNAs in glioma. In addition, the high heterogeneities among different types of gliomas lead to inconsistencies in the therapeutic response and prognosis of patients. But considerable commonalities in malignant biological behaviors also exist among different types of gliomas due to their shared origin from nerve epithelium-derived cells. We believed it was valuable and feasible to explore common biomarkers which could overcome the heterogeneities among different types of gliomas. Hence, we enrolled all types of gliomas in this study but didn’t focused on a particular type.

In this study, we identified 24 prognostic ferroptosis-related lncRNAs, 15 of which were selected to construct the prognostic FRLS. No matter for training cohort (TCGA) or validation cohorts (CGGA and Rembrandt), the FRLS showed robust capacity in predicting survival outcomes of glioma patients. Combining the prognostic FRLS with other independent prognostic factors (age and grade), a nomogram was established with improved predictive capacity of OS. PCA and functional enrichment analyses revealed the potential differences in in ferroptosis sensitivity between high- and low-risk groups. Immune-related biological processes and pathways were also observed in functional enrichment analyses. We further uncovered the differential immune landscape between the two risk subgroups by comparing the immune and stromal scores, abundance of immune cells, and expression levels of immunoregulatory molecules. Moreover, the FRLS-based risk stratification may herald the difference in efficacy of radiotherapy.

It is self-evident that the epigenetic dysregulation has been implicated in the PCD evasion of tumor cells. As an important component of epigenetics, lncRNAs have been intensively studied regarding its role in the regulation of classical PCD, such as apoptosis and autophagy (Rossi and Antonangeli, 2014; Sun, 2018; Bermúdez et al., 2019; Talebian et al., 2020; Wei et al., 2020; Zhang et al., 2020). Recently, the mysterious veil of lncRNAs in the ferroptosis process of tumors has been gradually uncovered. For example, Mao et al. (2018) demonstrated that a cytosolic lncRNA P53RRA promotes ferroptosis in lung cancer cells via activating p53 pathway. However, what we know about the role of lncRNAs in ferroptosis is just a tip of the iceberg. To our knowledge, signature based on ferroptosis-related lncRNAs has not yet been reported. In this study, the FRLS contained 15 ferroptosis-related lncRNAs, several of which have been confirmed to be correlated with tumor progression. For example, SNAI3-AS1 promotes PEG10-mediated proliferation and metastasis by acting as a sponge for miR-27-3p and miR-34a-5p in hepatocellular carcinoma (Liu J. et al., 2020). WDFY3-AS2 suppresses cell proliferation, migration, invasion, and epithelial-to-mesenchymal transition (EMT) via miR-18a/RORA axis in ovarian cancer (Zhou et al., 2020). MIR22HG acts as a critical inducer of the Wnt/β-catenin signal pathway to facilitate proliferation and invasion in GBM (Sun et al., 2020). However, reports on how these 15 lncRNAs participate in ferroptosis process have been even rarer. Only the lncRNA PVT1 has been reported to regulate the balance of iron metabolism in hepatocellular carcinoma via PVT1/miR-150/HIG2 axis (Xu et al., 2018), and regulate ferroptosis in acute ischemic stroke (AIS) through miR-214-mediated TFR1 and p53 expression (Lu et al., 2020). Our study investigated the prognostic value of these ferroptosis-related lncRNAs in glioma. Future in-depth experimental researches are warranted to explore their potential regulatory effects on the ferroptosis process.

Ferroptosis is also considered as immunogenic cell death, characterized by the release of damage associated molecular patterns (DAMPs) from dying tumor cells (Tang et al., 2019; Wen et al., 2019; Wan et al., 2020). Efimova et al. (2020) demonstrated that early ferroptotic glioma GL261 cells could promote the phenotypic maturation of bone-marrow derived dendritic cells (BMDCs) via the release of DAMPs, including ATP and HMGB1, and induce efficient antitumor immunity. In our study, the DEGs between different risk subgroups were enriched in many immune-related biological processes and pathways. Further analyses found that the high-risk group exhibited higher immune scores, higher abundance of immunosuppressive cells (Tregs, M2-type macrophages), and higher expression levels of immune checkpoints and macrophage associated molecules. In contrast, tumor killer cells (activated NK cells) showed a higher abundance in the low-risk group. These results suggested that the FRLS was correlated, to some extent, with the immune landscape of glioma microenvironment. However, the potential molecular mechanisms connecting the ferroptosis and glioma immunity remain to be further investigated. On the other hand, several studies have begun to explore the feasibility and effectiveness of combined cancer immunotherapy with ferroptosis inducers (Wang W. et al., 2019; Tang R. et al., 2020; Xu J. et al., 2020). Wang et al. first reported that CD8 + T cells activated by checkpoint blockade could enhance ferroptosis-specific lipid peroxidation in tumor cells through the release of interferon gamma (IFNγ) to downregulate the expression of SLC3A2 and SLC7A11 (Wang W. et al., 2019). In our study, patients in the high-risk group showed relatively higher expression levels of immune checkpoints. For these patients, targeting tumor-specific ferroptosis pathways, maybe some lncRNAs, is a promising regimen in combination with checkpoint blockade.

Currently, resistance to radiotherapy and TMZ treatment is an intractable problem in the management of glioma patients. Accumulating studies suggested that ferroptosis inducers are promising in the field of oncotherapy for their radiosensitizing and chemosensitizing effects. Chen et al. reported that erastin, a ferroptosis inducer, could improve the sensitivity of glioma cells to TMZ (Chen et al., 2015). Ye et al. revealed that ferroptosis inducers enhanced the efficacy of radiotherapy in human patient-derived models of glioma (Ye et al., 2020). In our study, the FRLS-based risk stratification was not correlated with the efficacy of TMZ treatment. There seems no difference in the survival of patients with or without TMZ treatment. The main reason might be that patients without TMZ treatment received other types of chemotherapy, such as PCV treatment. Interestingly, the FRLS-based risk stratification was correlated with the efficacy of radiotherapy. Patients in the low-risk group were more likely to obtain a survival benefit from radiotherapy than those in the high-risk group. This finding may promote the choice of individually therapeutic strategies.

Indubitably, some limitations must be addressed in this study. Firstly, these three cohorts had diverse degrees of deficiency in clinical information and the sample size of Rembrandt cohort was relatively small, resulting in insufficient validation of partial results. Secondly, the available samples for qRT-PCR were not sufficient and were not from the same zone in brain. More tissue samples will be needed in further work to make the results more solid. Thirdly, the FRLS was constructed and validated with retrospective data from public databases. Using prospective data to assess its clinical utility would be more convincing. Finally, the molecular mechanism has not been characterized, further experiments are essential to explore the interactions between the lncRNAs and ferroptosis-related genes.

To sum up, this study fills the gap of FRLS in prognostic prediction of glioma. The prognostic FRLS constructed in our study exhibited robust capacity in predicting survival outcomes of glioma patients, and was correlated with immune landscape of glioma microenvironment. The FRLS-based risk stratification was indicative of different efficacy of radiotherapy to a certain extent. We hope that these findings will offer some useful insights for subsequent studies and clinical practice.

## Data Availability Statement

Publicly available datasets were analyzed in this study. This data can be found here: The data analyzed in this study can be acquired in the TCGA (https://portal.gdc.cancer.gov/), CGGA (http://www.cgga.org.cn/) and Rembrandt (http://gliovis.bioinfo.cnio.es/) websites.

## Ethics Statement

The studies involving human participants were reviewed and approved by The Medical Ethics Committee of Union Hospital, Tongji Medical College, Huazhong University of Science and Technology. The patients/participants provided their written informed consent to participate in this study. Written informed consent was obtained from the individual(s) for the publication of any potentially identifiable images or data included in this article.

## Author Contributions

JZ and ZZ constructed this study. JZ, ZZ, YQ, MW, HY, and ZW performed the data analysis, figures plotted, and writing. XW and XJ were responsible for the critical reading of the manuscript. All authors contributed to the article and approved the submitted version.

## Conflict of Interest

The authors declare that the research was conducted in the absence of any commercial or financial relationships that could be construed as a potential conflict of interest.

## Footnotes

1 https://portal.gdc.cancer.gov/

2 http://www.cgga.org.cn/

3 http://gliovis.bioinfo.cnio.es/

4 https://www.gencodegenes.org/human/

## Supplementary Material

Supplementary Figure 1 **(A–O)** The Kaplan–Meier curves of TCGA cohort showing that patients with different expression levels of the 15 ferroptosis-related lncRNAs had different overall survival.

Supplementary Figure 2 **(A,B)** The expression levels of the 15 ferroptosis-related lncRNAs between samples with different WHO grades in the TCGA and CGGA cohorts. **p* < 0.05, ***p* < 0.01, and ****p* < 0.001.

Supplementary Figure 3 Correlation analysis between the prognostic FRLS and clinicopathological characteristics in the CGGA and Rembrandt cohorts. **(A–G)** Different levels of risk scores in glioma patients stratified by age, gender, 2016 WHO classification, grade, IDH status, 1p19q codeletion and MGMT promoter status in the CGGA cohort. **(H–K)** Different levels of risk scores in glioma patients stratified by age, gender and grade in the Rembrandt cohort. A, astrocytoma; AA, anaplastic astrocytoma; O, oligodendroglioma; AO, anaplastic oligodendroglioma; GBM, glioblastoma. **p* < 0.05, ***p* < 0.01, ****p* < 0.001, and ^*ns*^ No significance.

Supplementary Figure 4 The Kaplan-Meier curves for subgroup survival in the TCGA **(A)**, CGGA **(B)**, and Rembrandt **(C)** cohorts.

Supplementary Figure 5 Evaluation of the nomogram in the CCGA cohort. **(A–C)** Calibration curves showing the concordance between predicted and observed 1-, 3-, and 5-year overall survival (OS). **(D–F)** The receiver operating characteristic (ROC) curve analyses of the nomogram in in predicting 1-, 3-, and 5-year OS.

Supplementary Figure 6 The correlation between the risk scores and the expression levels of gene markers of immune cell in the TCGA cohort, including B cell **(A)**, T cell **(B)**, CD8 + T cell **(C)**, Treg cell **(D)**, NK cell **(E)**, M1 macrophage **(F)**, M2 macrophage **(G)**, and neutrophils **(H)**.

Supplementary Figure 7 Correlation of the prognostic FRLS with the immune landscape of glioma microenvironment in the CGGA cohort. **(A–D)** Comparison of immune scores, stromal scores, ESTIMATE scores and tumor purity between the high- and low-risk groups. **(E)** The abundance of 22 immune cells in the high- and low-risk groups. A total of 394 patients with CIBERSORT *p* ≥ 0.05 were excluded. A total of 657 patients with CIBERSORT *p* ≥ 0.05 were excluded. **(F)** The expression levels of immune checkpoints and macrophage associated molecules in the high- and low-risk groups. **p* < 0.05, ***p* < 0.01, and ****p* < 0.001.

Supplementary Figure 8 The correlation between FRLS-based risk stratification and the efficacy of TMZ treatment in the CGGA cohort. **(A)** The Kaplan-Meier curves for patients with or without TMZ treatment in the high- and low-risk groups. **(B–E)** The Kaplan-Meier curves for WHO grade II **(B)**, WHO grade III **(C)**, GBM **(D)**, and MGMT promoter methylated **(E)** patients with or without TMZ treatment in the high- and low-risk groups.

Supplementary Figure 9 The correlation between FRLS-based risk stratification and the efficacy of TMZ treatment in the TCGA cohort. **(A)** The Kaplan-Meier curves for patients with or without TMZ treatment in the high- and low-risk groups. **(B–E)** The Kaplan-Meier curves for WHO grade II **(B)**, WHO grade III **(C)**, GBM **(D)**, and MGMT promoter methylated **(E)** patients with or without TMZ treatment in the high- and low-risk groups. All GBM patients with available data were in the high-risk group.

Supplementary Figure 10 The correlation between FRLS-based risk stratification and the efficacy of radiotherapy in the TCGA cohort. **(A)** The Kaplan-Meier curves for patients with or without radiotherapy in the high- and low-risk groups. **(B–E)** The Kaplan-Meier curves for WHO grade II **(B)**, WHO grade III **(C)**, MGMT promoter unmethylated **(D)**, and MGMT promoter methylated **(E)** patients with or without radiotherapy in the high- and low-risk groups.
