# Supplementary figures and images for: A Prognostic Ferroptosis-Related lncRNAs Signature Associated With Immune Landscape and Radiotherapy Response in Glioma
Source: Front Cell Dev Biol. 2021 May 19;9:675555. doi: 10.3389/fcell.2021.675555 (PMC8170051; doi:10.3389/fcell.2021.675555)

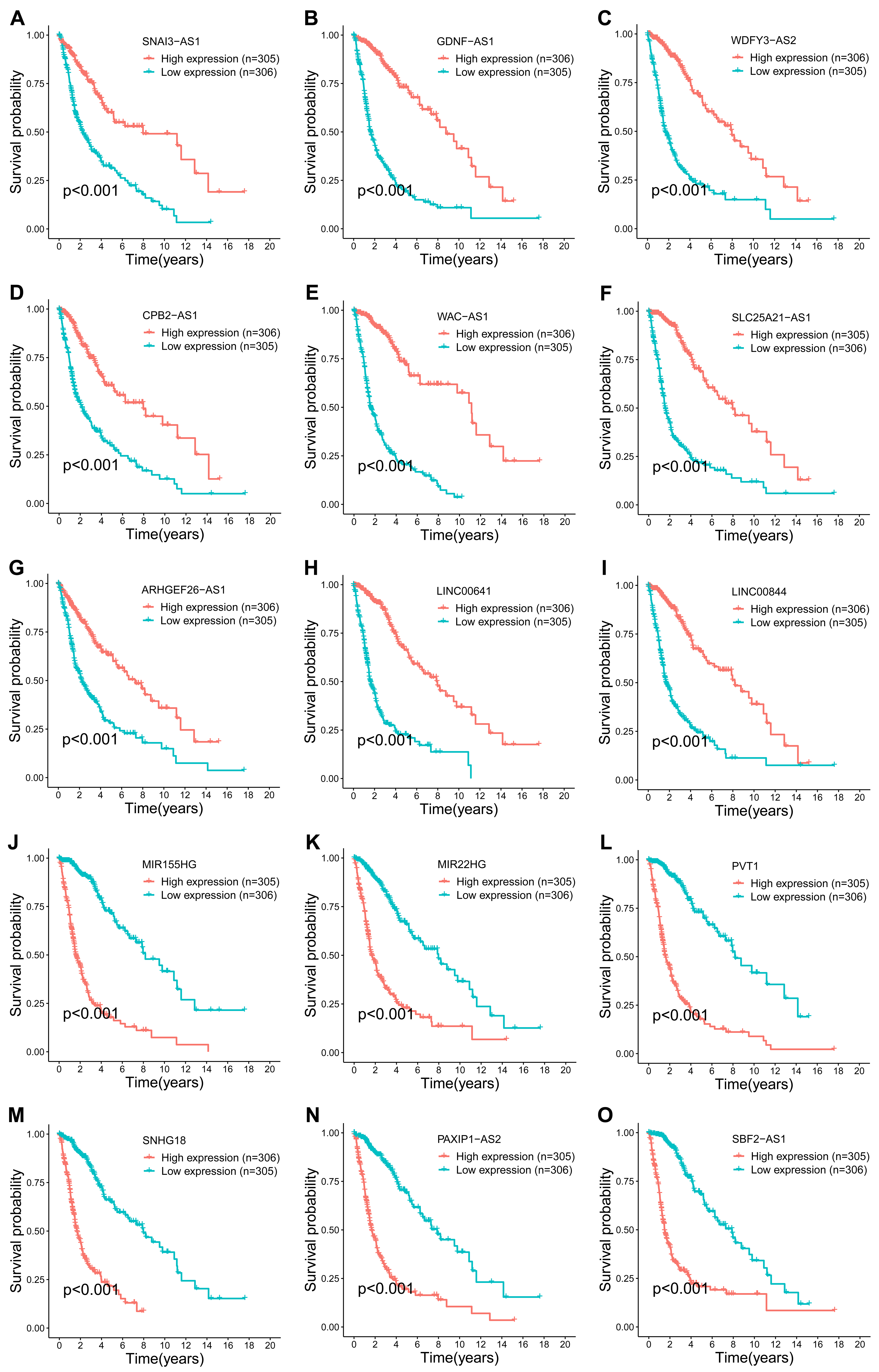

Supplement: Supplementary Figure 1 — (A–O) The Kaplan–Meier curves of TCGA cohort showing that patients with different expression levels of the 15 ferroptosis-related lncRNAs had different overall survival. [file Image_1.TIF]

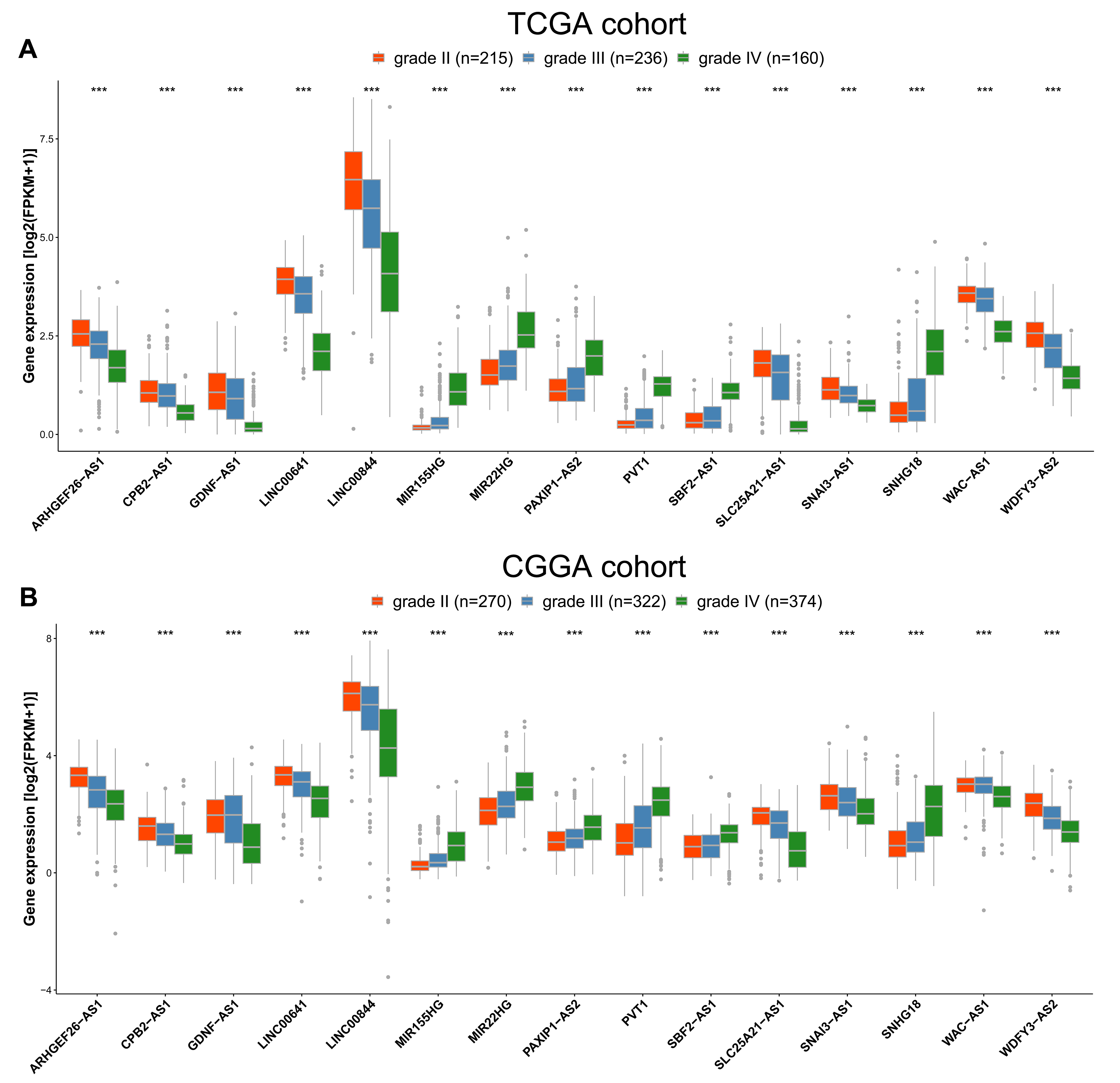

Supplement: Supplementary Figure 2 — (A,B) The expression levels of the 15 ferroptosis-related lncRNAs between samples with different WHO grades in the TCGA and CGGA cohorts. *p < 0.05, **p < 0.01, and ***p < 0.001. [file Image_2.TIF]

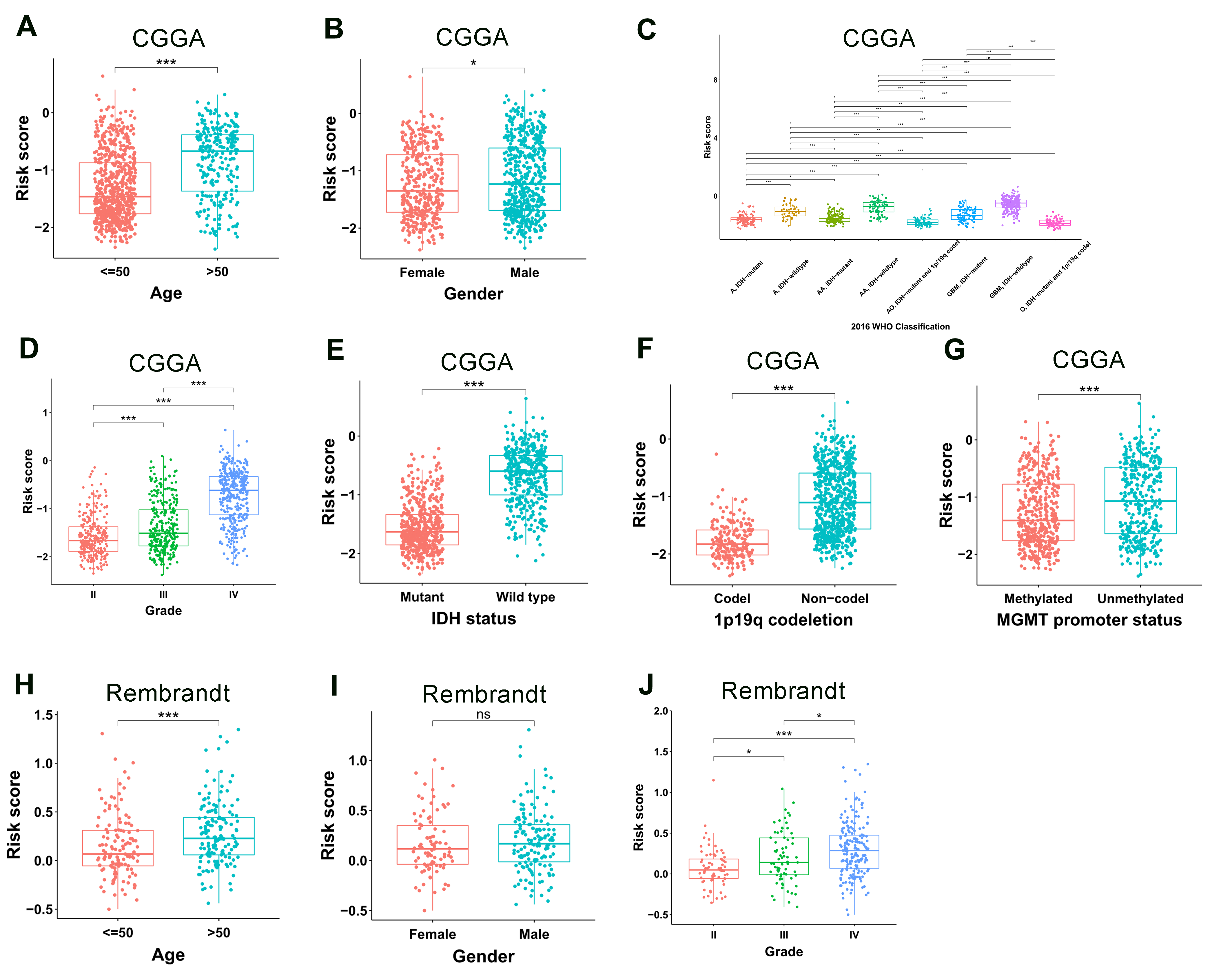

Supplement: Supplementary Figure 3 — Correlation analysis between the prognostic FRLS and clinicopathological characteristics in the CGGA and Rembrandt cohorts. (A–G) Different levels of risk scores in glioma patients stratified by age, gender, 2016 WHO classification, grade, IDH status, 1p19q codeletion and MGMT promoter status in the CGGA cohort. (H–K) Different levels of risk scores in glioma patients stratified by age, gender and grade in the Rembrandt cohort. A, astrocytoma; AA, anaplastic astrocytoma; O, oligodendroglioma; AO, anaplastic oligodendroglioma; GBM, glioblastoma. *p < 0.05, **p < 0.01, ***p < 0.001, and ns No significance. [file Image_3.TIF]

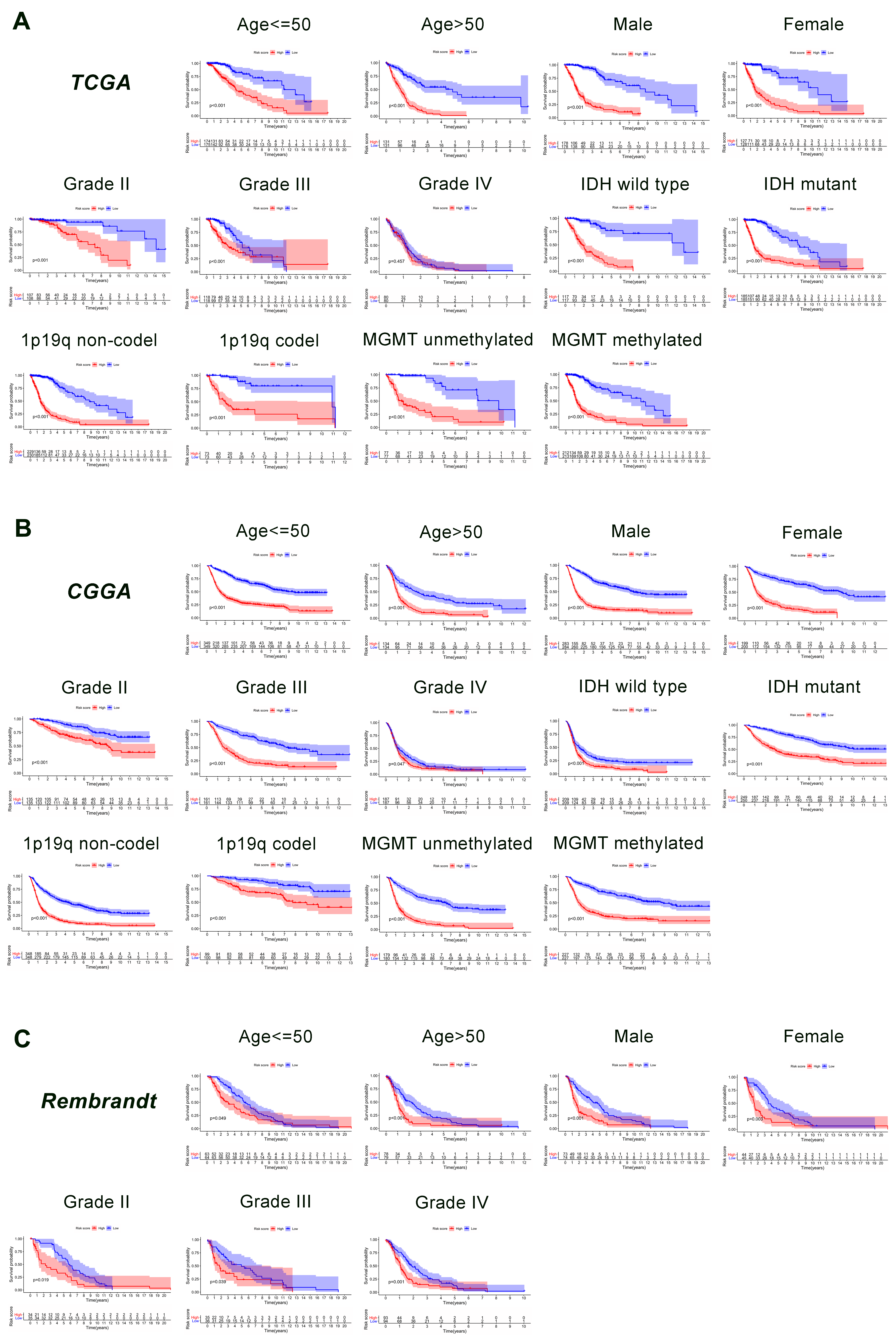

Supplement: Supplementary Figure 4 — The Kaplan-Meier curves for subgroup survival in the TCGA (A), CGGA (B), and Rembrandt (C) cohorts. [file Image_4.TIF]

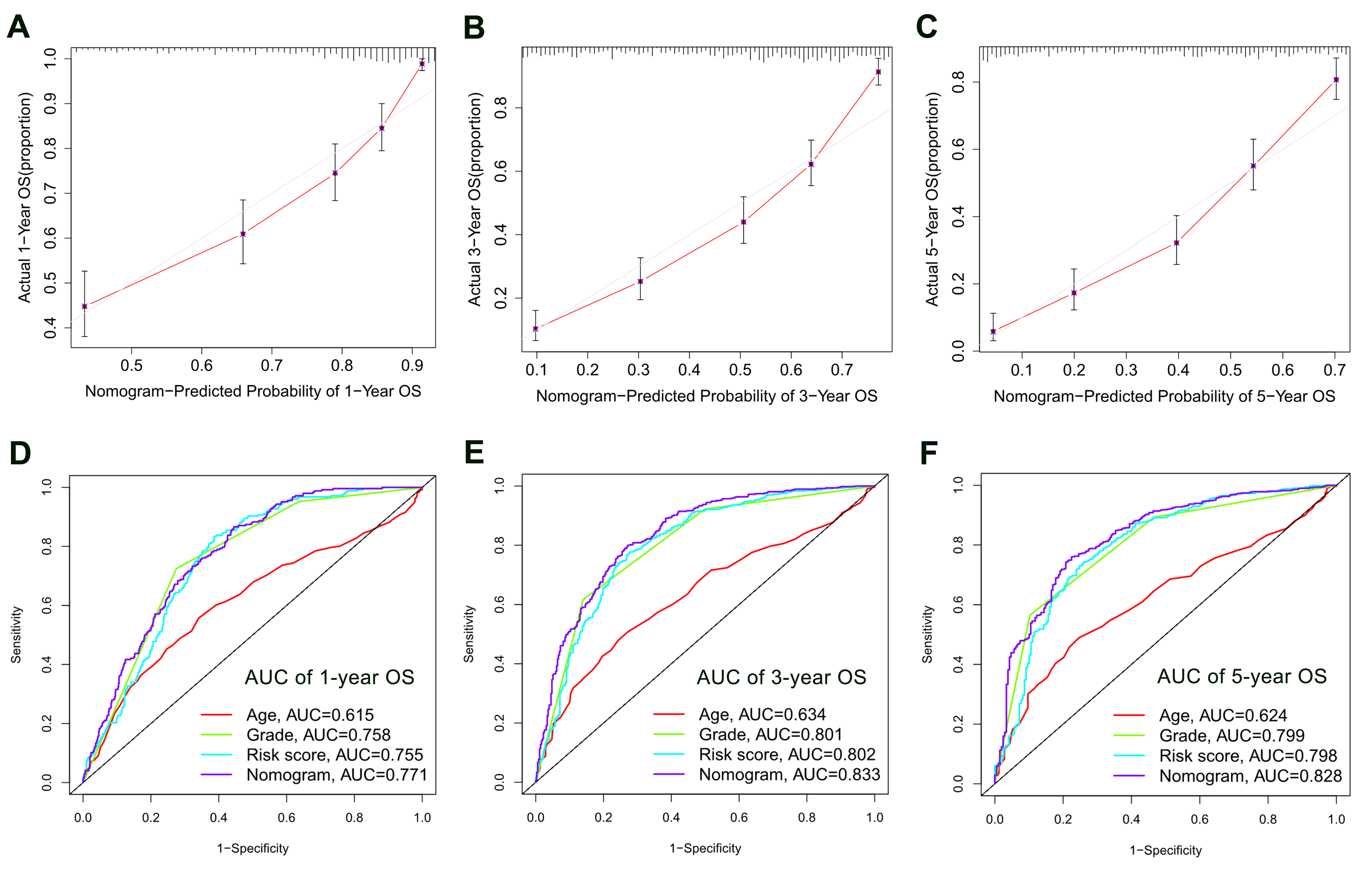

Supplement: Supplementary Figure 5 — Evaluation of the nomogram in the CCGA cohort. (A–C) Calibration curves showing the concordance between predicted and observed 1-, 3-, and 5-year overall survival (OS). (D–F) The receiver operating characteristic (ROC) curve analyses of the nomogram in in predicting 1-, 3-, and 5-year OS. [file Image_5.TIF]

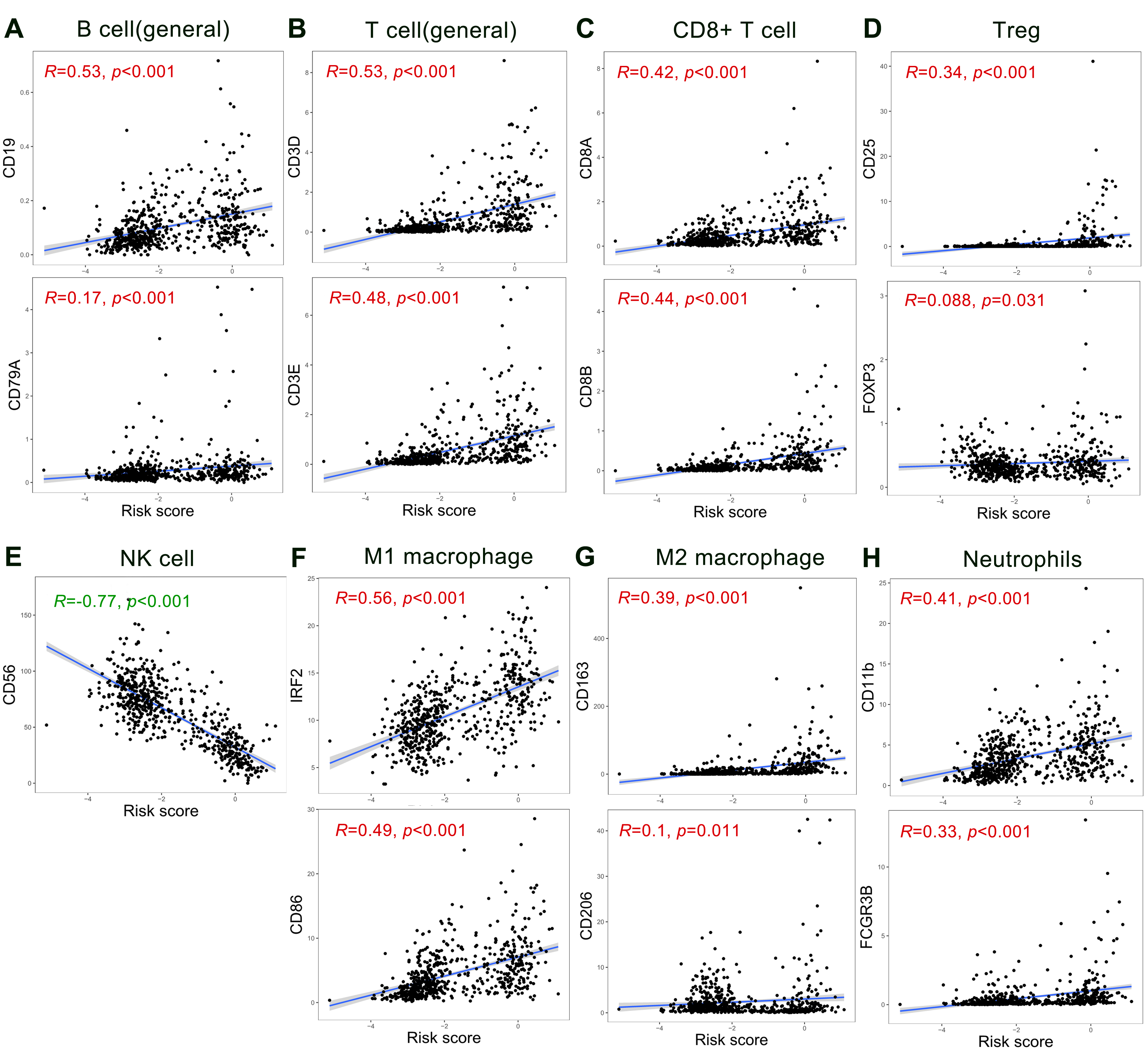

Supplement: Supplementary Figure 6 — The correlation between the risk scores and the expression levels of gene markers of immune cell in the TCGA cohort, including B cell (A), T cell (B), CD8 + T cell (C), Treg cell (D), NK cell (E), M1 macrophage (F), M2 macrophage (G), and neutrophils (H). [file Image_6.TIF]

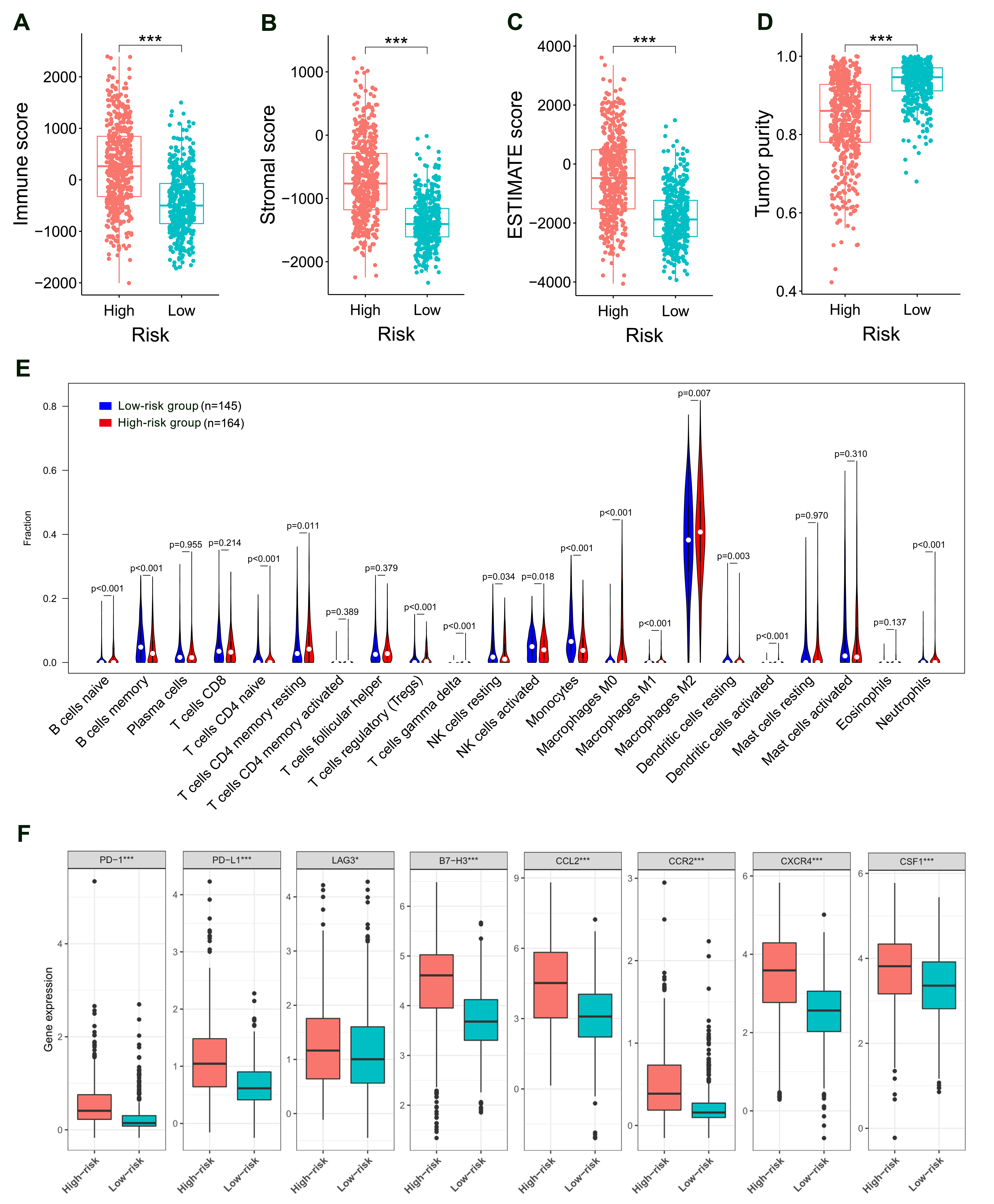

Supplement: Supplementary Figure 7 — Correlation of the prognostic FRLS with the immune landscape of glioma microenvironment in the CGGA cohort. (A–D) Comparison of immune scores, stromal scores, ESTIMATE scores and tumor purity between the high- and low-risk groups. (E) The abundance of 22 immune cells in the high- and low-risk groups. A total of 394 patients with CIBERSORT p ≥ 0.05 were excluded. A total of 657 patients with CIBERSORT p ≥ 0.05 were excluded. (F) The expression levels of immune checkpoints and macrophage associated molecules in the high- and low-risk groups. *p < 0.05, **p < 0.01, and ***p < 0.001. [file Image_7.TIF]

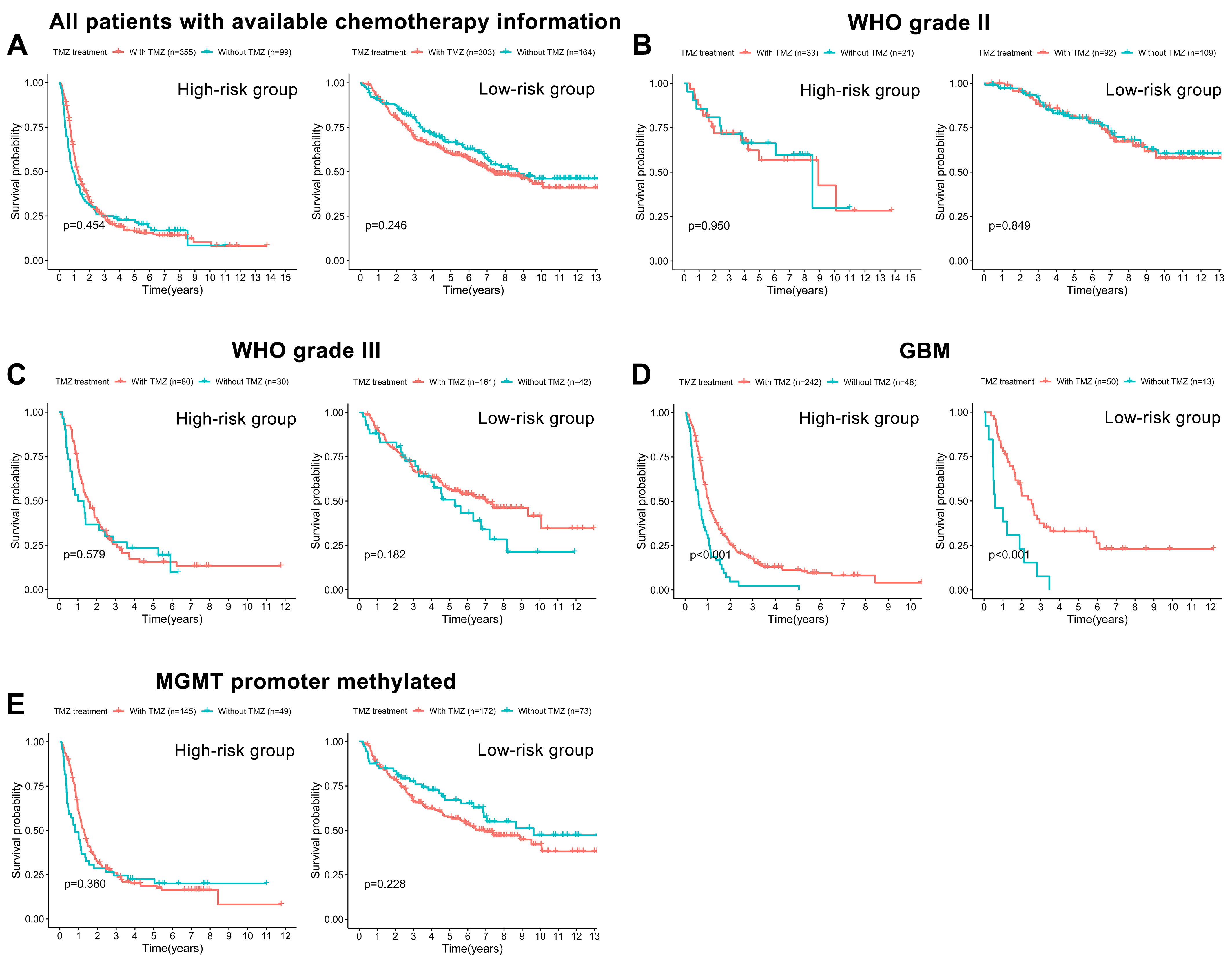

Supplement: Supplementary Figure 8 — The correlation between FRLS-based risk stratification and the efficacy of TMZ treatment in the CGGA cohort. (A) The Kaplan-Meier curves for patients with or without TMZ treatment in the high- and low-risk groups. (B–E) The Kaplan-Meier curves for WHO grade II (B), WHO grade III (C), GBM (D), and MGMT promoter methylated (E) patients with or without TMZ treatment in the high- and low-risk groups. [file Image_8.TIF]

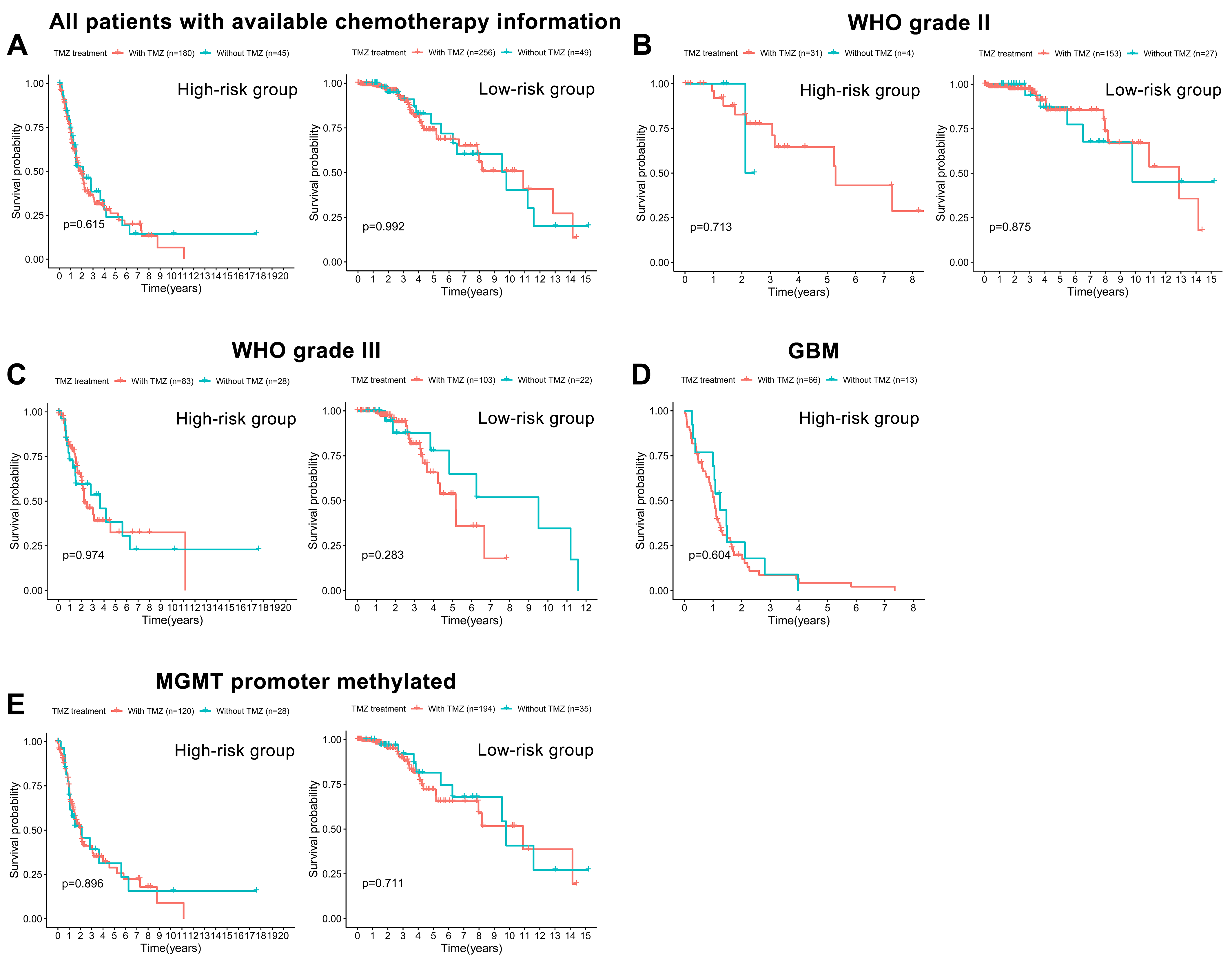

Supplement: Supplementary Figure 9 — The correlation between FRLS-based risk stratification and the efficacy of TMZ treatment in the TCGA cohort. (A) The Kaplan-Meier curves for patients with or without TMZ treatment in the high- and low-risk groups. (B–E) The Kaplan-Meier curves for WHO grade II (B), WHO grade III (C), GBM (D), and MGMT promoter methylated (E) patients with or without TMZ treatment in the high- and low-risk groups. All GBM patients with available data were in the high-risk group. [file Image_9.TIF]

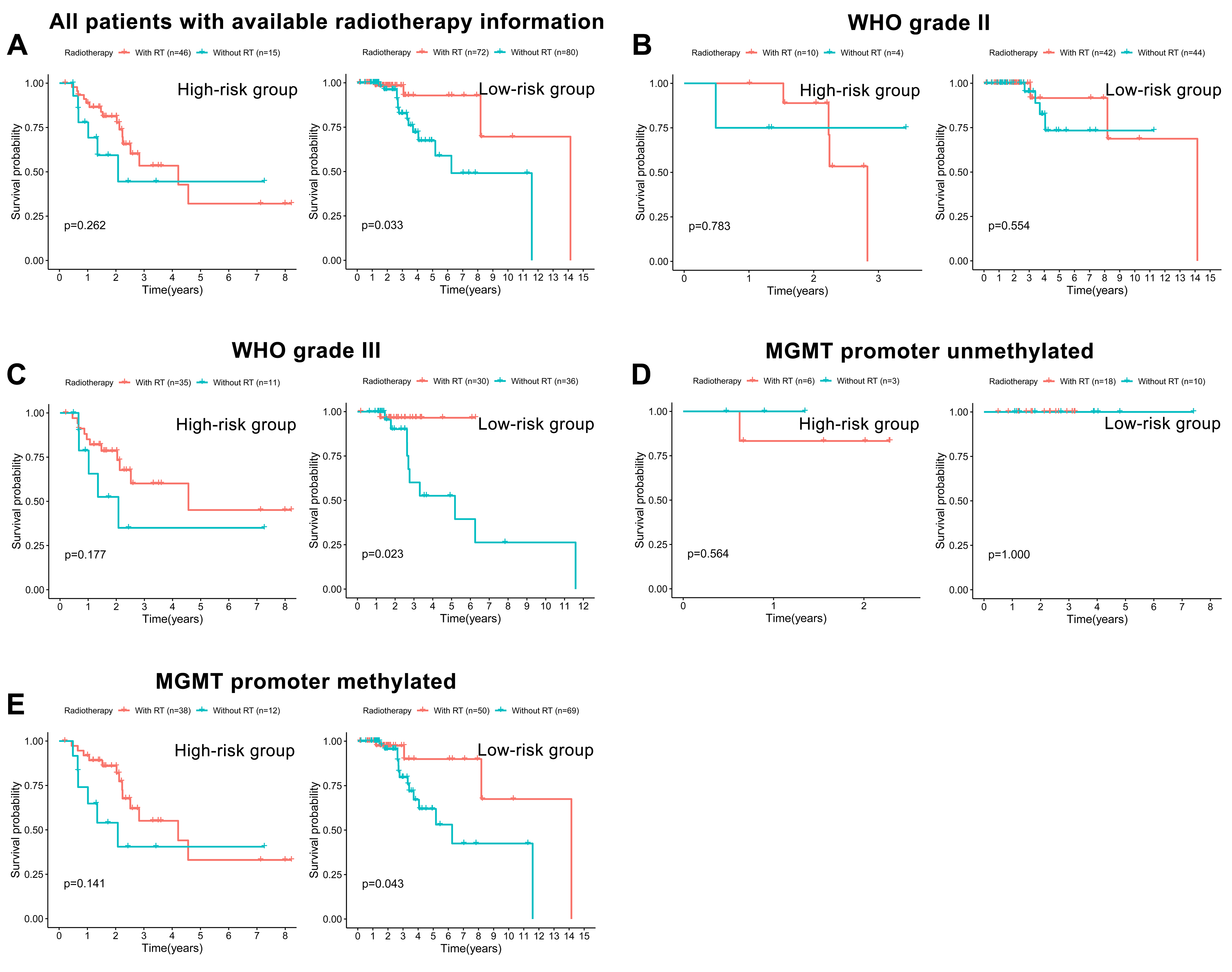

Supplement: Supplementary Figure 10 — The correlation between FRLS-based risk stratification and the efficacy of radiotherapy in the TCGA cohort. (A) The Kaplan-Meier curves for patients with or without radiotherapy in the high- and low-risk groups. (B–E) The Kaplan-Meier curves for WHO grade II (B), WHO grade III (C), MGMT promoter unmethylated (D), and MGMT promoter methylated (E) patients with or without radiotherapy in the high- and low-risk groups. [file Image_10.TIF]
