# Supplementary material for: A Prognostic Ferroptosis-Related lncRNAs Signature Associated With Immune Landscape and Radiotherapy Response in Glioma
Source: Front Cell Dev Biol. 2021 May 19;9:675555. doi: 10.3389/fcell.2021.675555 (PMC8170051; doi:10.3389/fcell.2021.675555)
Supplement: Supplementary file 11 [file Data_Sheet_1.docx]

**Supplementary Table S1. The location of tissues used for qRT-PCR.**

| **Location** | **Nontumor brain tissues** | **WHO grade II-III glioma tissues** | **GBM tissues** | **Total** |
| --- | --- | --- | --- | --- |
| frontal lobe | 4 | 4 | 2 | 10 |
| temporal lobe | 2 | 0 | 1 | 3 |
| parietal lobe | 0 | 1 | 1 | 2 |
| occipital lobe | 0 | 0 | 0 | 0 |
| insular lobe | 0 | 1 | 0 | 1 |
| Total | 6 | 6 | 4 | 16 |

**Supplementary Table S2. The Primers sequence used in this study**

| **Name** | **Forward-primer** | **Reverse-primer** |
| --- | --- | --- |
| SNAI3-AS1 | 5’-GCGTTATGTCGTTTGGTTGATG-3’ | 5’-TGGCAGGAATGAGGTGAGC-3’ |
| GDNF-AS1 | 5’-AACA GGCAAACACAAGGTGC-3’ | 5’-GCTTGCAGTGT GATGTTGGG-3’ |
| WDFY3-AS2 | 5’-TTGGGGCACTCATCCCATTC-3’ | 5’-TTGAGCTCGGACTGTGCATT-3’ |
| CPB2-AS1 | 5’-GCCTAGTAGGGGGACTTCCA-3’ | 5’-TCCATCCTTCCCCGCTAAGA-3’ |
| SBF2-AS1 | 5’-CACGACCCAGAAGGAGTCTAC-3’ | 5’-CCCGGTACCTTCCTGTCATA-3’ |
| PAXIP1-AS2 | 5’-TGACCGAACGCTGAATGTGA-3’ | 5’-CTCCTGTGAACCACAGCACA-3’ |
| SNHG18 | 5’-TGTGGCAGCCCACTCTATTG-3’ | 5’-TGGTGGACTTGAGTGGAAGC-3’ |
| PVT1 | 5’-GGGGAATAACGCTGGTGGAA-3’ | 5’-CCCATGGACATCCAAGCTGT-3’ |
| GAPDH | 5’-AAAAGCATCACCCGGAGGAGAA-3’ | 5’-AAGGAAATGAATGGGCAGCCG-3’ |

**Supplementary Table S3. The comparison of clinicopathological characteristics between the high-risk and low-risk groups in the TCGA cohort**

| **Clinicopathological characteristics** | **High-risk group**  **(n = 305)** | **Low-risk group**  **(n = 306)** | ***p-*value** |
| --- | --- | --- | --- |
| **Age** (Mean ± SD) | 53.57 ± 15.52 | 41.35 ± 12.35 | <0.001 |
| **Gender** |  |  | 0.127 |
| Male | 187 (61.3%) | 169 (55.2%) |  |
| Female | 118 (38.7%) | 187 (44.8%) |  |
| **Histology** |  |  | <0.001 |
| A, IDH-mutant | 9 (3.6%) | 30 (24.2%) |  |
| A, IDH-wildtype | 5 (2.0%) | 10 (8.1%) |  |
| AA, IDH-mutant | 51 (20.5%) | 27 (21.8%) |  |
| AA, IDH-wildtype | 19 (7.6%) | 14 (11.3%) |  |
| O, IDH-mutant and 1p/19q-codel | 1 (0.4%) | 28 (22.6%) |  |
| AO, IDH-mutant and 1p/19q-codel | 8 (3.2%) | 14 (11.3%) |  |
| GBM, IDH-mutant | 68 (27.3%) | 1 (0.8%) |  |
| GBM, IDH-wildtype | 88 (35.4%) | 0 (0.0%) |  |
| **WHO grade** |  |  | <0.001 |
| II | 35 (11.5%) | 180 (58.8%) |  |
| III | 111 (36.4%) | 125 (40.9%) |  |
| IV | 159 (52.1%) | 1 (0.3%) |  |
| **IDH status** |  |  | 0.001 |
| Mutant | 164 (54.7%) | 206 (67.8%) |  |
| Wild type | 136 (45.3%) | 98 (32.2%) |  |
| **1p/19q codeletion** |  |  | 0.027 |
| Codel | 61 (20.3%) | 85 (28.0%) |  |
| Non-codel | 240 (79.7%) | 219 (72.0%) |  |
| **MGMT promoter status** |  |  | 0.008 |
| Methylated | 195(68.4%) | 230 (78.2%) |  |
| Unmethylated | 90 (31.6%) | 64 (21.8%) |  |

A, astrocytoma; AA, anaplastic astrocytoma; O, oligodendroglioma; AO, anaplastic oligodendroglioma; GBM, glioblastoma

**Supplementary Table S4. The comparison of clinicopathological characteristics between the high-risk and low-risk groups in the CGGA cohort**

| **Clinicopathological characteristics** | **High-risk group**  **(n = 483)** | **Low-risk group**  **(n = 483)** | ***p-*value** |
| --- | --- | --- | --- |
| **Age** (Mean ± SD) | 46.10 ± 14.24 | 40.64 ± 9.34 | <0.001 |
| **Gender** |  |  | 0.267 |
| Male | 292 (60.5%) | 275 (56.9%) |  |
| Female | 191 (39.5%) | 208 (43.1%) |  |
| **Histology** |  |  | <0.001 |
| A, IDH-mutant | 16 (3.6%) | 94 (22.1%) |  |
| A, IDH-wildtype | 29 (6.5%) | 14 (3.3%) |  |
| AA, IDH-mutant | 28 (6.3%) | 93 (21.9%) |  |
| AA, IDH-wildtype | 65 (14.6%) | 17 (4.0%) |  |
| O, IDH-mutant and 1p/19q codel | 1 (0.2%) | 83 (19.5%) |  |
| AO, IDH-mutant and 1p/19q codel | 3 (0.7%) | 64 (15.1%) |  |
| GBM, IDH-mutant | 39 (8.7%) | 45 (10.6%) |  |
| GBM, IDH-wildtype | 265 (59.4%) | 15 (3.5%) |  |
| **WHO grade** |  |  | <0.001 |
| II | 58 (12.0%) | 212 (43.9%) |  |
| III | 116 (24.0%) | 206 (42.7%) |  |
| IV | 309 (64.0%) | 65 (13.4%) |  |
| **IDH status** |  |  | <0.001 |
| Mutant | 109 (22.9%) | 390 (88.4%) |  |
| Wild type | 367 (77.1%) | 51 (11.6%) |  |
| **1p/19q codeletion** |  |  | <0.001 |
| Codel | 12 (2.9%) | 187 (39.2%) |  |
| Non-codel | 406 (97.1%) | 290 (60.8%) |  |
| **MGMT promoter status** |  |  | <0.001 |
| Methylated | 201 (48.9%) | 253 (62.9%) |  |
| Unmethylated | 210 (51.1%) | 149 (37.1%) |  |

A, astrocytoma; AA, anaplastic astrocytoma; O, oligodendroglioma; AO, anaplastic oligodendroglioma; GBM, glioblastoma

**Supplementary Table S5. The comparison of clinicopathological characteristics between the high-risk and low-risk groups in the Rembrandt cohort**

| **Clinicopathological characteristics** | **High-risk group**  **(n = 164)** | **Low-risk group**  **(n = 163)** | ***p-*value** |
| --- | --- | --- | --- |
| **Age** |  |  | 0.002 |
| <=50 | 47 (35.1%) | 80 (53.3%) |  |
| >50 | 87 (64.9%) | 70 (46.7%) |  |
| **Gender** |  |  | 0.064 |
| Male | 71 (68.9%) | 76 (57.1%) |  |
| Female | 32 (31.1%) | 57 (42.9%) |  |
| **WHO grade** |  |  | <0.001 |
| II | 18 (11.0%) | 51 (31.3%) |  |
| III | 33 (20.1%) | 38 (23.3%) |  |
| IV | 113 (68.9%) | 74 (45.4%) |  |

**Supplementary Table S6. The FPLS-based risk score adjusted by other clinicopathological characteristics**

| **Adjusted variable** | **HR (95% CI) of Risk score** | ***p*-value** |
| --- | --- | --- |
| *TCGA* |  |  |
| **Age** (Continuous) | 2.440 (2,119-2.810) | <0.001 |
| **Gender** (Male vs Female) | 2.986 (2.632-3.387) | <0.001 |
| **Grade** (Continuous: IV, III and II) | 2.372 (1.940-2.901) | <0.001 |
| **IDH** (Mutant vs wild type) | 2.973 (2.620-3.374) | <0.001 |
| **1p/19q** (Codel vs Non-codel) | 2.990 (2.636-3.391) | <0.001 |
| **MGMT** (Methylated vs Unmethylated) | 2.971 (2.618-3.372) | <0.001 |
| *CGGA* |  |  |
| **Age** (Continuous) | 2.925 (2.564-3.336) | <0.001 |
| **Gender** (Male vs Female) | 3.120 (2.743-3.548) | <0.001 |
| **Grade** (Continuous: IV, III and II) | 1.996 (1.721-2.315) | <0.001 |
| **IDH** (Mutant vs wild type) | 2.662 (2.217-3.196) | <0.001 |
| **1p/19q** (Codel vs Non-codel) | 2.711 (2.343-3.136) | <0.001 |
| **MGMT** (Methylated vs Unmethylated) | 2.996 (2.614-3.434) | <0.001 |
| *Rembrandt* |  |  |
| **Gender** (Male vs Female) | 3.807 (2.441-5.937) | <0.001 |
| **Grade** (Continuous: IV, III and II) | 2.430 (1.650-3.579) | <0.001 |
